# Supplementary material for: Graph-based autoencoder integrates spatial transcriptomics with chromatin images and identifies joint biomarkers for Alzheimer’s disease
Source: Nat Commun. 2022 Dec 3;13:7480. doi: 10.1038/s41467-022-35233-1 (PMC9719477; doi:10.1038/s41467-022-35233-1)
Supplement: Supplementary file 1 — Supplementary information [file 41467_2022_35233_MOESM1_ESM.pdf]

Supplementary Information:

Integration of Spatial Transcriptomics with Chromatin Images Using Graph-Based Autoencoder Identifies Joint Biomarkers for Alzheimer's Disease

Xinyi Zhang[1,2], Xiao Wang[1,2], GV Shivashankar[3,4], Caroline Uhler[1,2,*]

[1] Massachusetts Institute of Technology, U.S.A.
[2] Broad Institute of MIT and Harvard, U.S.A.
[3] ETH Zurich, Switzerland
[4] Paul Scherrer Institute, Switzerland
* To whom correspondence should be addressed; E-mail: cuhler@mit.edu

This PDF file includes:

Supplementary Figures 1-29

Supplementary References

Supplementary Information:

Integration of Spatial Transcriptomics with Chromatin Images Using Graph-Based Autoencoder Identifies Joint Biomarkers for Alzheimer's Disease

Xinyi Zhang[1,2], Xiao Wang[1,2], GV Shivashankar[3,4], Caroline Uhler[1,2,*]

[1] Massachusetts Institute of Technology, U.S.A.
[2] Broad Institute of MIT and Harvard, U.S.A.
[3] ETH Zurich, Switzerland
[4] Paul Scherrer Institute, Switzerland
* To whom correspondence should be addressed; E-mail: cuhler@mit.edu

This PDF file includes:

Supplementary Figures 1-29

Supplementary References

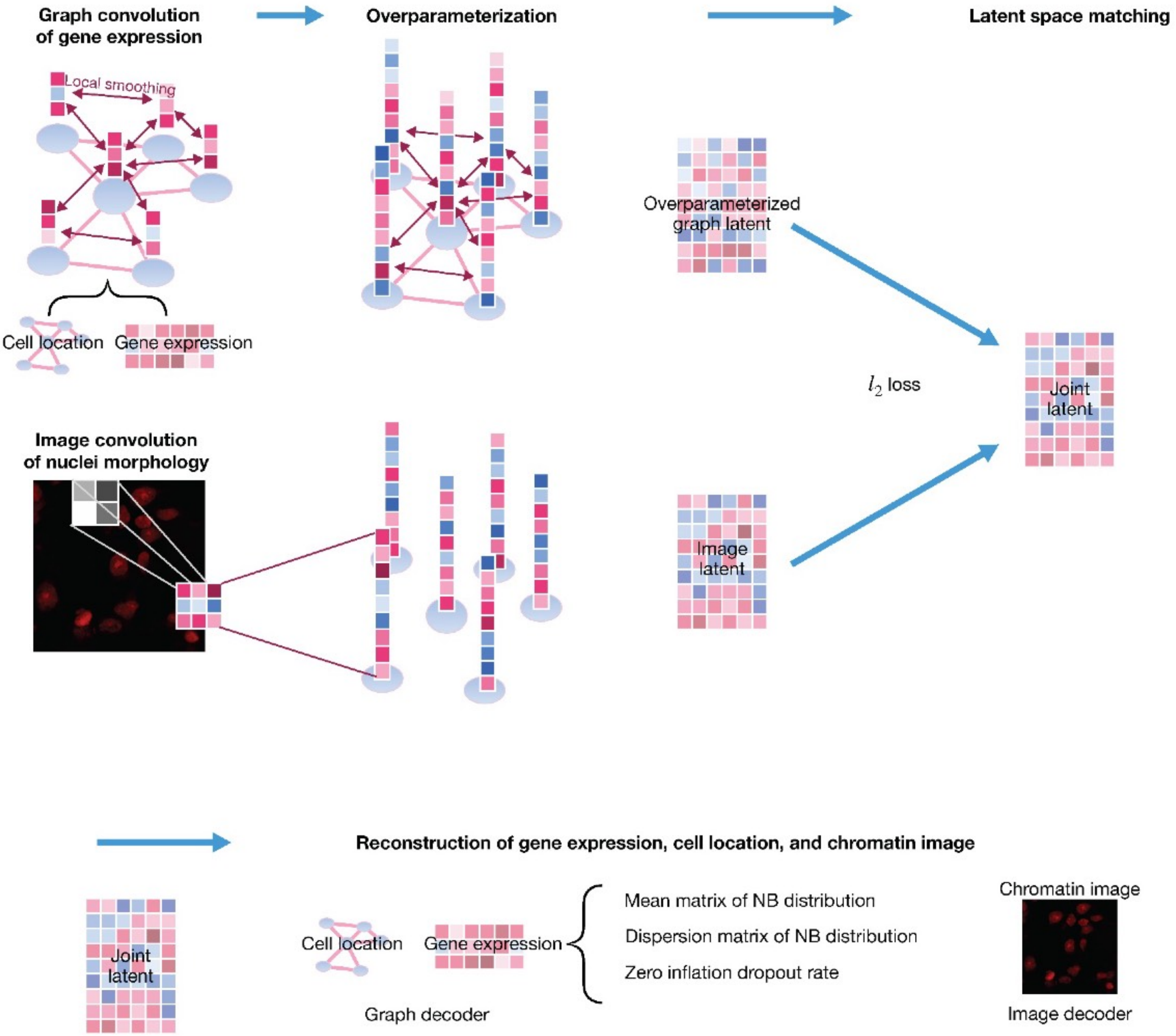

Supplementary Figure 1

Supplementary Figure 1. A detailed schematic of STACI.

A graph is constructed for each tissue sample where each node is a cell and the node feature is the gene expression profile of the cell. Cell adjacencies are represented by an adjacency matrix. The graph convolutional layer performs a local smoothing operation before passing through a linear layer and leaky ReLU activation (see Methods). Hidden layers of the graph autoencoder are over-parameterized with dimensions larger than the input dimension to correct for batch effects. Cell adjacencies are decoded by the inner product decoder for the adjacency matrix. Gene expression decoder estimates the mean, dispersion, and dropout rate in a zero-inflated negative binomial distribution that maximizes the likelihood of the input data. Images are encoded to the latent space through a CNN autoencoder. The latent space of the CNN autoencoder is matched with the latent space of the graph autoencoder by l_2 loss.

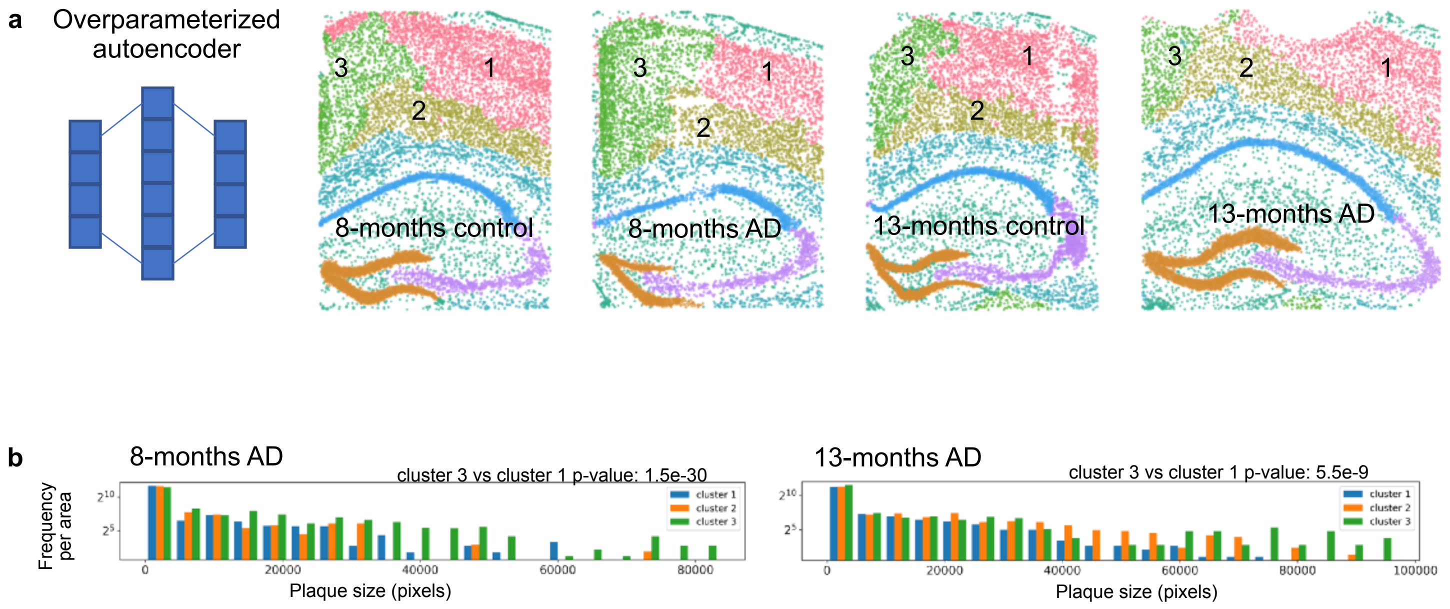

Supplementary Figure 2

Supplementary Figure 2. Validation of STACI on four held-out mouse samples shows consistent tissue segmentation and plaque size distribution in the cortex.

a) The full STACI model with 6000 latent dimensions was applied to the four samples held-out in the original analysis. All eight samples were clustered using Leiden with a resolution of 0.82.
b) Histograms of plaque size, measured in number of pixels, are plotted for the cortex regions of the two AD samples. Frequency is normalized by the area of each cortex region. Consistent with the findings in the original four mice samples, larger plaque sizes are observed in cluster 3 as compared to cluster 1 in the 8-month held-out AD mouse (p-values: 1.5e-30 for cluster 3 vs cluster 1) and are observed in both cluster 2 and cluster 3 as compared to cluster 1 in the held-out 13-month AD mouse (p-values: 5.5e-9 for cluster 3 vs cluster 1 and 6.4e-15 for cluster 2 vs cluster 1). Two-sided T-tests were used to obtain the raw p-values.

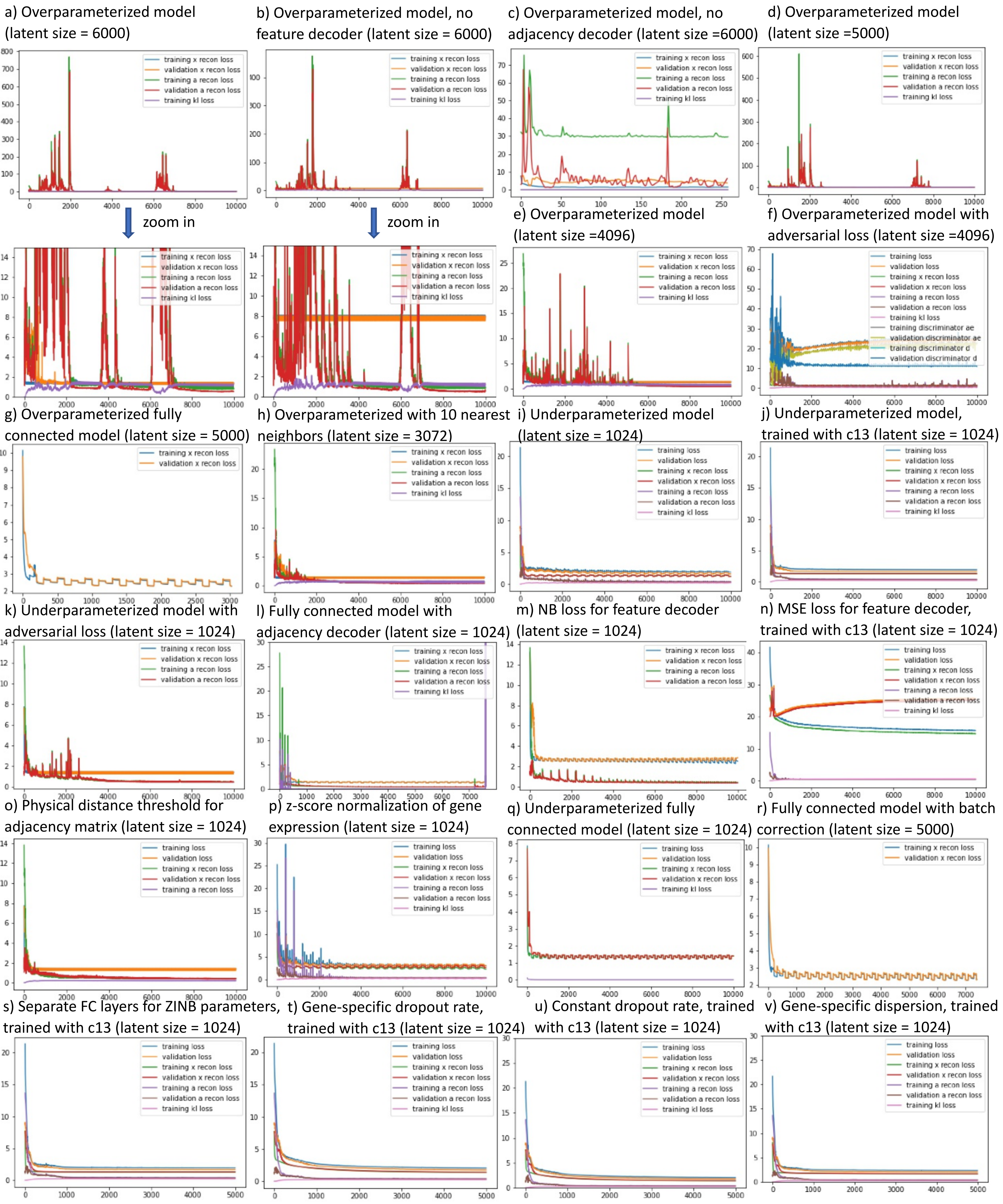

Supplementary Figure 3

Supplementary Figure 3. Training and validation loss curves.

Unless otherwise noted, all gene expression profiles are normalized within each cell by min-max scaling after log2 transformation, and the input adjacency matrix is defined using the 20 nearest neighbors measured in Euclidean distance. a) Graph convolutional autoencoder, trained with all four samples. b) Graph convolutional autoencoder without feature decoder, trained with all four samples. c) Graph convolutional autoencoder without adjacency decoder, trained with all four samples. d)-e) Graph convolutional autoencoder, trained with all four samples. f) Graph convolutional autoencoder with additional adversarial loss in the latent space for batch correction, trained with all four samples. g) Autoencoder with fully connected layers and no adjacency reconstruction loss, trained with all four samples. h) Graph convolutional autoencoder, trained with all four samples. Adjacency matrix is defined by 10 nearest neighbors measured in Euclidean distance. i) Graph convolutional autoencoder, trained with all four samples. j) Graph convolutional autoencoder, trained on 13-months control sample. k) Graph convolutional autoencoder with additional adversarial loss in the latent space for batch correction, trained with all four samples. l) Autoencoder with fully connected layers and with adjacency reconstruction loss, trained with all four samples. m) Graph convolutional autoencoder with negative binomial loss for the feature decoder, i.e. without dropout term, trained with all four samples. n) Graph convolutional autoencoder, trained on 13-months control sample. Feature decoder reconstructs the input gene expression matrix directly. Mean squared error loss for the feature decoder is used. o) Graph convolutional autoencoder with physical distance threshold for the adjacency matrix, trained with all four samples. The maximum distance between two neighboring cells in each sample is 8 times the minimum cell to cell distance in the sample (approximately 36.4 µm in the 13-montsh control sample). p) Graph convolutional autoencoder, trained with all four samples. Gene expression is normalized by z-score normalization of each gene. q) Autoencoder with fully connected layers and no adjacency reconstruction loss, trained with all four samples. r) Autoencoder with fully connected layers and no adjacency reconstruction loss, trained with all four samples. Gene expression is normalized by z-score normalization of each gene and batch corrected as in Zeng *et al.*[1] s) Graph convolutional autoencoder, trained with the 13-months control sample. Instead of a shared linear layer between the latent representation and the decoder layers for each parameter, separate linear layers are used for each parameter. t) Graph convolutional autoencoder, trained with the 13-months control sample. A single dropout rate is inferred for each gene in each sample, instead of the cell by gene matrix of dropout rate as in the previous models. u) Graph convolutional autoencoder, trained with the 13-months control sample. A single dropout rate is inferred for each sample. v) Graph convolutional autoencoder, trained with the 13-months control sample. A single dispersion value for the ZINB model is inferred for each gene in each sample, instead of a dispersion matrix over all genes as in the previous models.

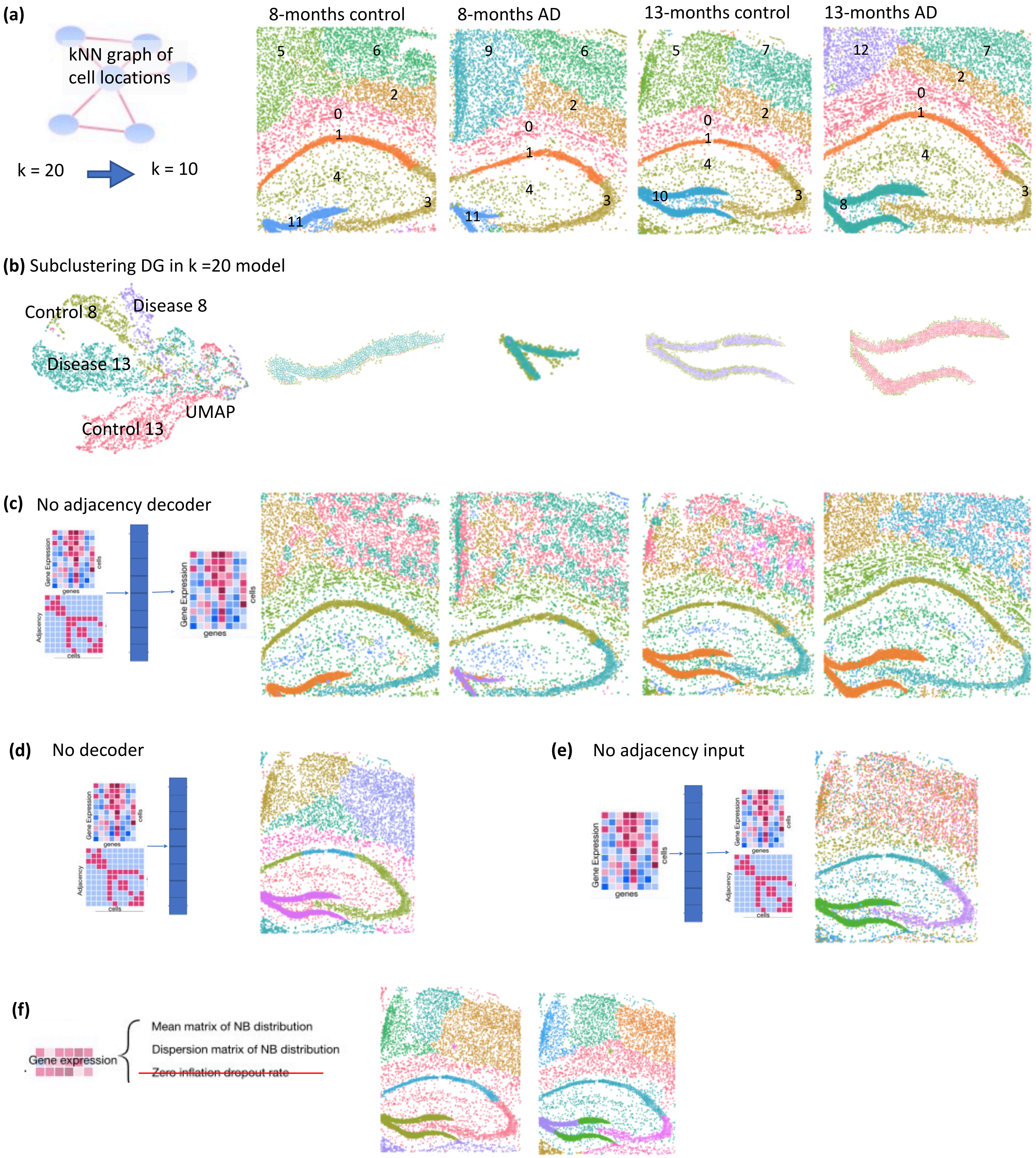

Supplementary Figure 4

Supplementary Figure 4. Comparisons of the clustering results of our full model to different variants of our model.

a) Graph convolutional autoencoder, trained with all four samples (full model). Adjacency matrix is defined using the 10 nearest neighbors measured in Euclidean distance. Latent size is 3072. Cells are colored and numbered by cluster identity.
b) Subclustering of the DG cluster in the full model. Graph convolutional autoencoder, trained with all four samples. Adjacency matrix is defined using the 20 nearest neighbors measured in Euclidean distance. Latent size is 6000. UMAP: cells are colored by tissue samples. Plots with cells in physical coordinates are colored by cluster identity.
c) Graph convolutional autoencoder without adjacency decoder, trained with all four samples. Adjacency matrix is defined using the 20 nearest neighbors measured in Euclidean distance. Latent size is 6000. Cells are colored by cluster identity.
d) Two local smoothing operations (i.e. convolutions) of the graph without training of the autoencoder. In each smoothing operation, each node is updated by the weighted average of its neighbors and itself, with weights defined by the adjacency matrix.
e) Fully connected encoder with both the adjacency decoder and the feature decoder, trained with all four samples. Adjacency matrix is defined using the 20 nearest neighbors measured in Euclidean distance. Latent space size is 1024.
f) Graph convolutional autoencoder with the feature decoder modeling a negative binomial distribution without zero inflation. Adjacency matrix is defined using the 20 nearest neighbors measured in Euclidean distance. Latent space size is 1024.

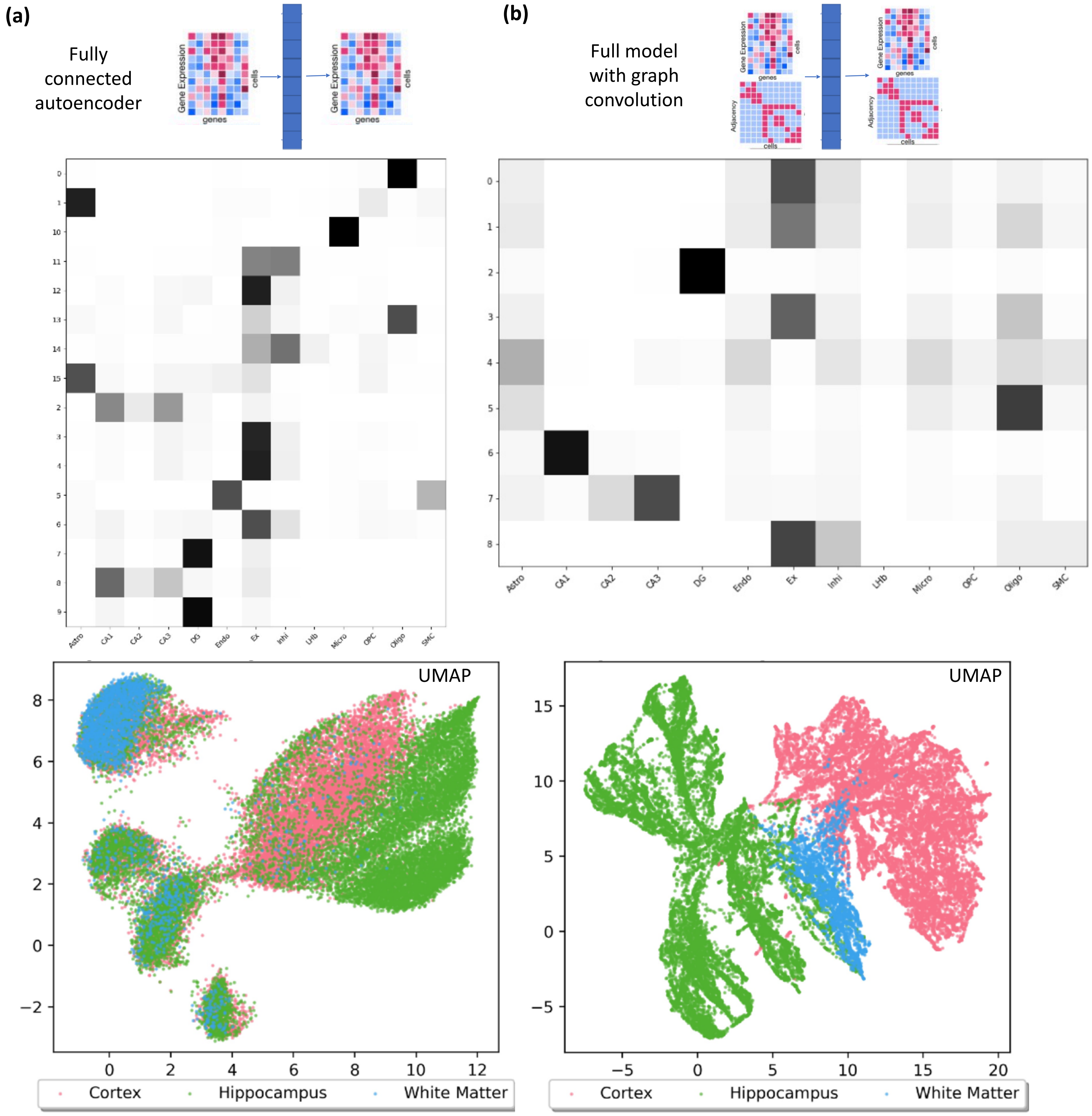

Supplementary Figure 5

Supplementary Figure 5. Cell type composition and UMAPs of clusters of latent representations. Cell type composition of clusters of latent representations in (a) autoencoder model with fully connected encoder and without using the adjacency matrix; (b) full graph convolutional autoencoder model. Each row is normalized to sum to 1. Input gene expression was normalized by min-max scaling of log2 transformed counts. Both models are over-parameterized with latent sizes of 5000 (a) and 6000 (b).

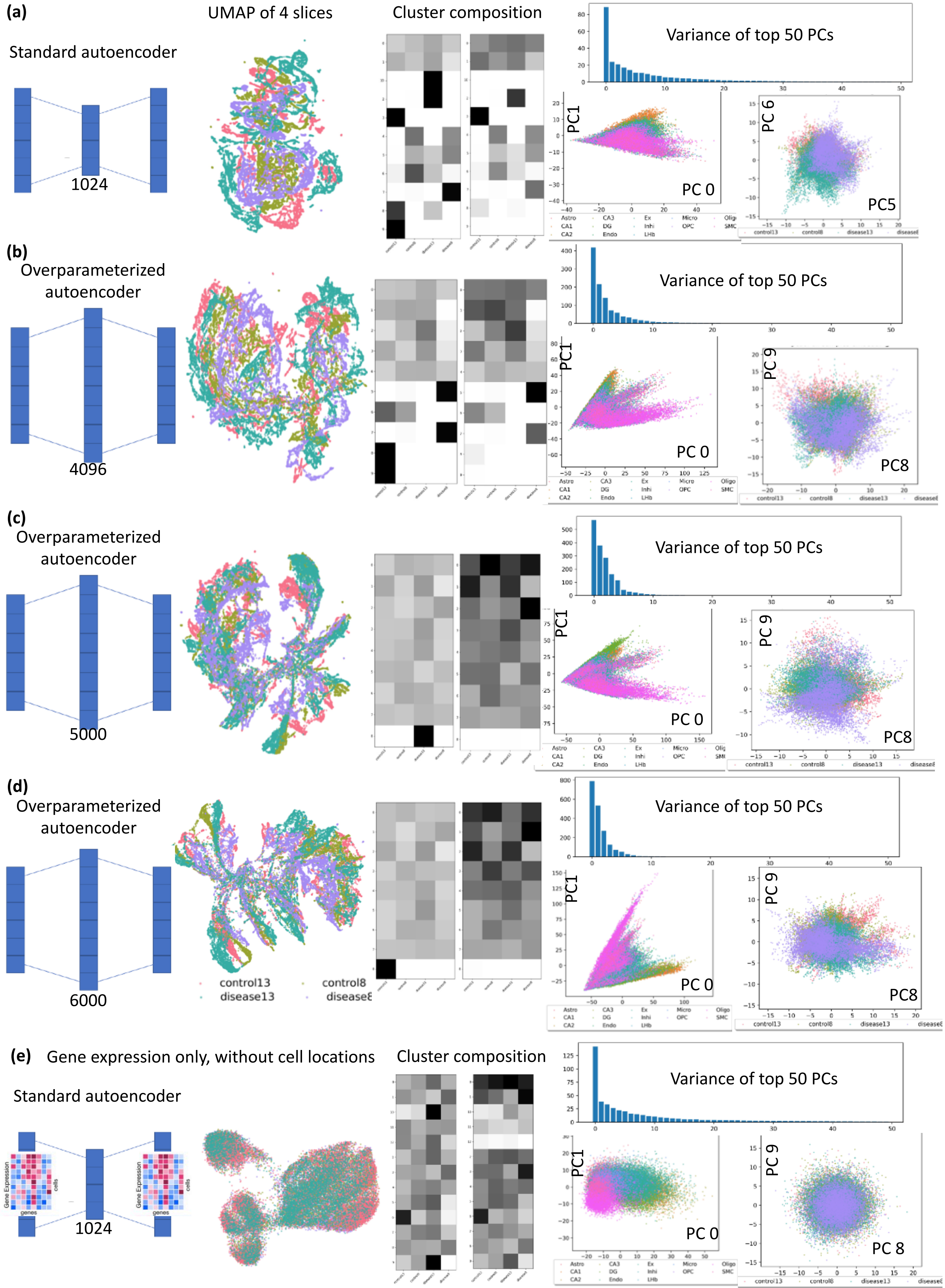

Supplementary Figure 6

Supplementary Figure 6. Over-parameterization corrects for batch effects by stretching the data along the direction of the top principal components.

Left: autoencoder schematic with the latent dimension size. UMAPs: latent representations colored by tissue samples. Cluster composition: number of cells in each cluster and each tissue sample normalized by rows (left) or columns (right) to sum up to 1 in the rows or columns. Darker color indicates higher fraction. Right: (top) Variance of the latent representations explained by the top 60 principal components; (bottom) The first principal components that lead to separation of cells by cell types (left) and tissue samples (right).

a)-d) Graph convolutional autoencoders with both gene expression and cell location as the input and the output (full model). e) Fully connected encoder with only gene expression as the input and the output.

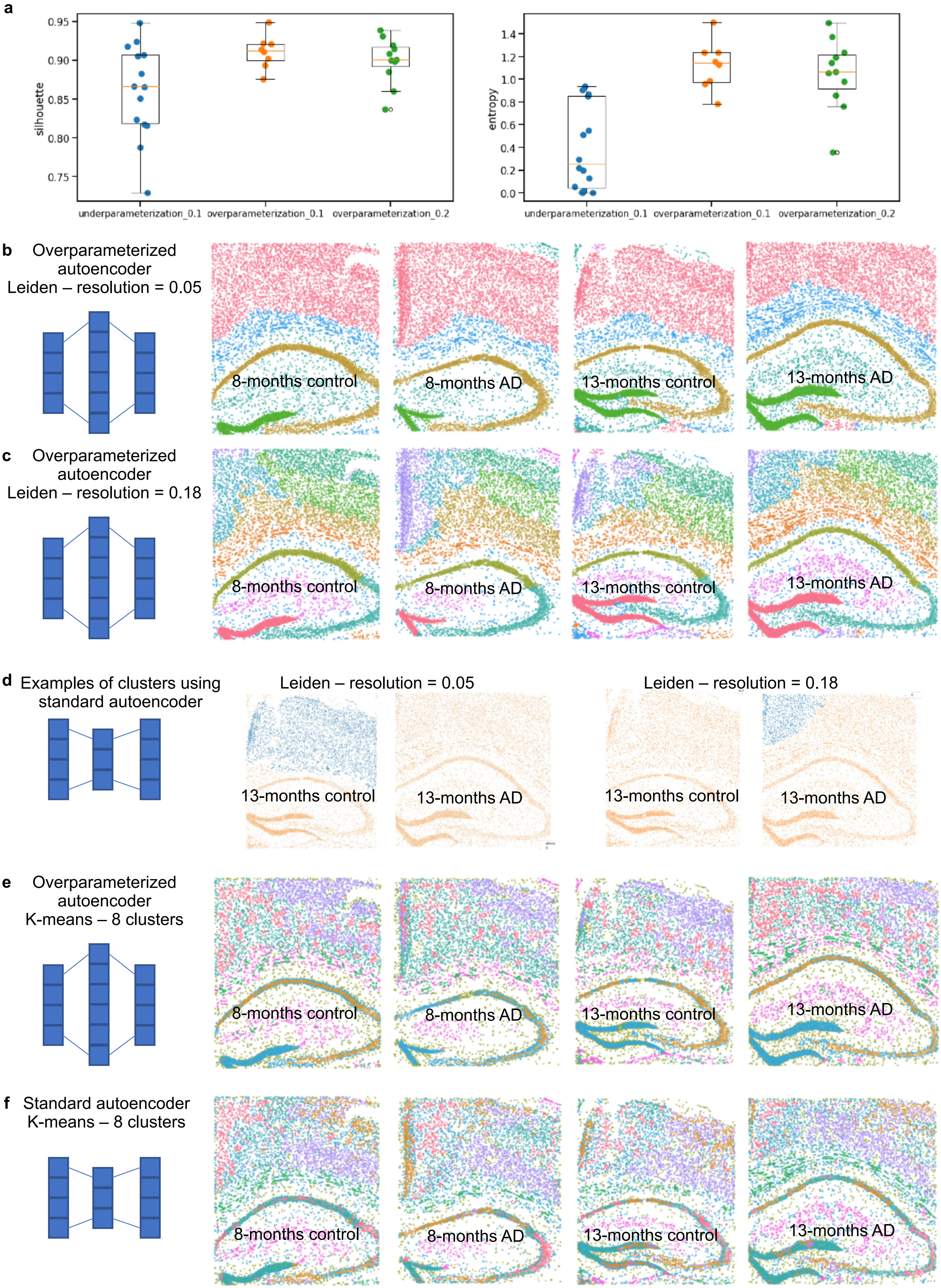

Supplementary Figure 7

Supplementary Figure 7. Over-parameterization consistently removes batch effects with different clustering methods and parameters.

a) Quantification of mixing of cells from different STARmap samples in the under-parameterized latent space and the over-parameterized latent space for each cluster. Each dot represents the score of one cluster. The numbers (0.1 or 0.2) indicate the clustering resolutions while all other clustering parameters are kept the same. Left: average silhouette width. Right: entropy of mixing. For both metrics, higher score means better mixing and less batch effects. Cells were from four mice with at least 7257 cells each. Bounds of boxes in the boxplots indicate quartiles. Whiskers extend from the bounds of boxes by 1.5 times the interquartile range. Outlier dots are those that pass the end of the whiskers.
b) Leiden clustering of the latent space of our over-parameterized model with a latent dimension of 6000 and clustering resolution of 0.05. Each dot is a cell plotted with its physical coordinates in the tissue and colored by the cluster identity. 20 nearest neighbor graph is used as the adjacency.
c) Leiden clustering of the same model as in b) with a clustering resolution of 0.18.
d) Cells from the same cluster (blue) using our model without over-parameterization correspond to different regions in 13-month control (left) and 13-month AD (right). The latent dimension of this model is 1024. The results of two different clustering resolutions are shown.
e) k-means clustering of the over-parameterized model in b) also results in consistent separation of all samples into different cortex regions, CA, and DG.
f) k-means clustering of the under-parameterized model in d) results in some cells from the cortex, CA, and DG being grouped into the same cluster.

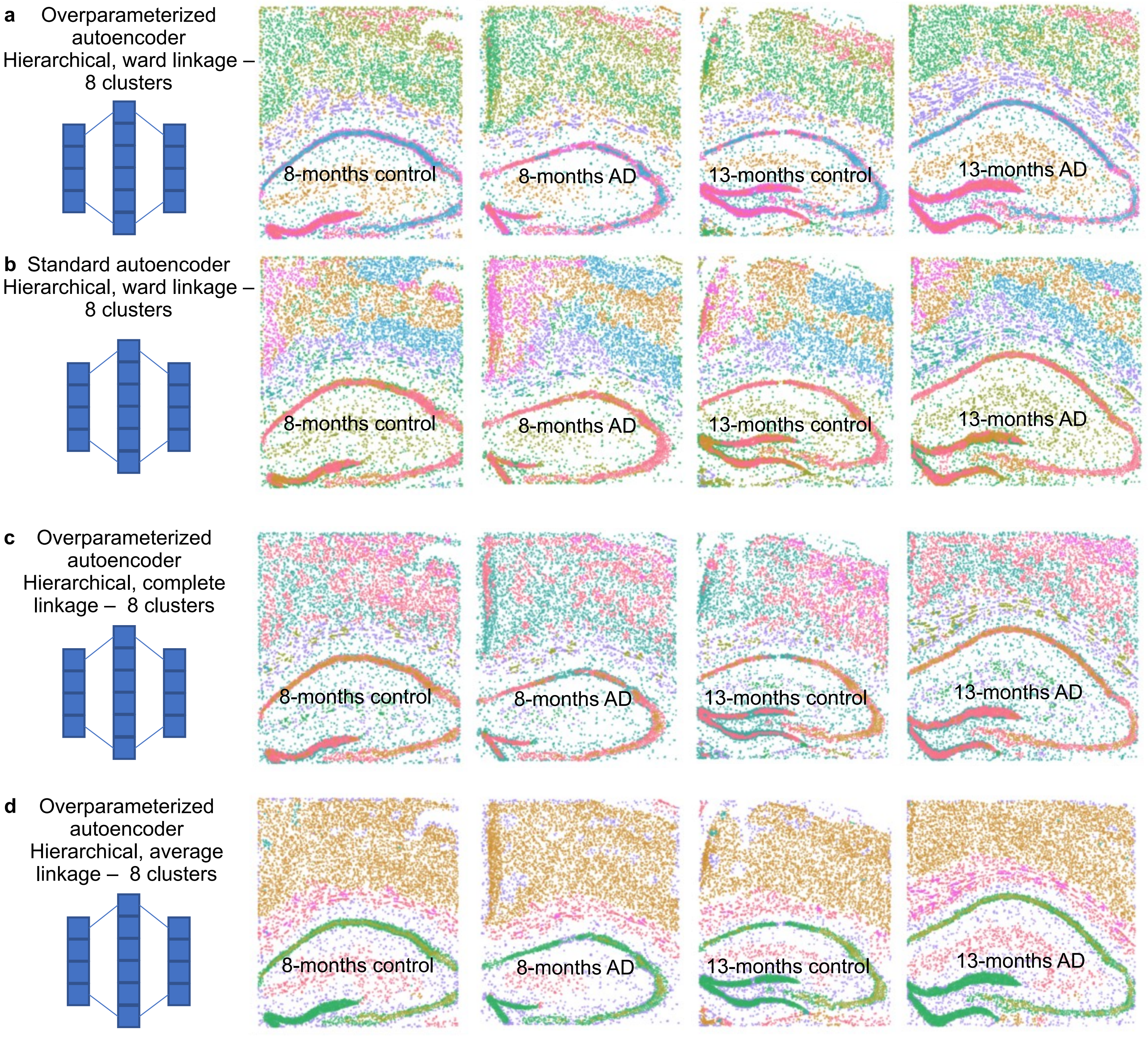

Supplementary Figure 8

Supplementary Figure 8. Over-parameterization consistently removes batch effects using hierarchical clustering with different linkages.

a) Hierarchical clustering of the latent space of our over-parameterized model with a latent dimension of 6000 and ward linkage. Each dot is a cell plotted with its physical coordinates in the tissue and colored by the cluster identity. 20 nearest neighbor graph is used as the adjacency.
b) Hierarchical clustering of the latent space of our under-parameterized model with a latent dimension of 1024 and ward linkage. Each dot is a cell plotted with its physical coordinates in the tissue and colored by the cluster identity. 20 nearest neighbor graph is used as the adjacency. The under-parameterized model is less consistent with the known anatomical regions and results in some cells of the cortex and DG in the same cluster.
c) Hierarchical clustering of the same model as in a) with complete linkage.
d) Hierarchical clustering of the same model as in a) with average linkage.

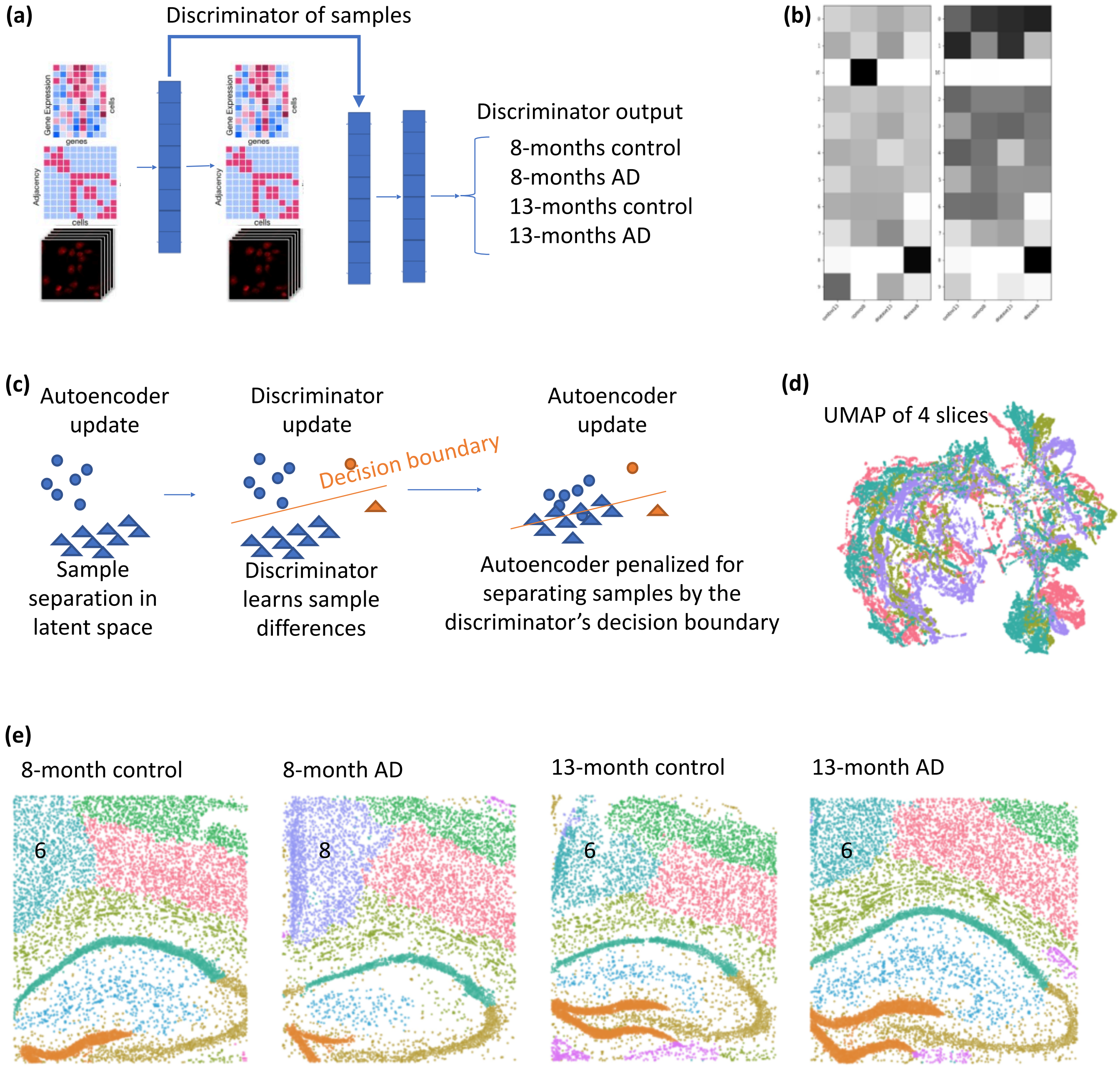

Supplementary Figure 9

Supplementary Figure 9. Batch correction with adversarial loss in the latent space.

a) A discriminator is trained to distinguish which tissue sample the input latent representation belongs to. b) Number of cells in each cluster and each tissue sample normalized by rows (left) and columns (right). Darker color indicates higher fraction. Latent dimension is 1024. c) Schematic of the training procedure which alternates between autoencoder update and discriminator update at each epoch. The discriminator was trained to correctly classify cells by their tissue samples. Autoencoder was trained to confuse the discriminator by assigning equal probability to each tissue sample. d) UMAP of the latent representation of the same model as in b). e) Cells plotted by physical coordinates in tissue and colored by Leiden clusters.

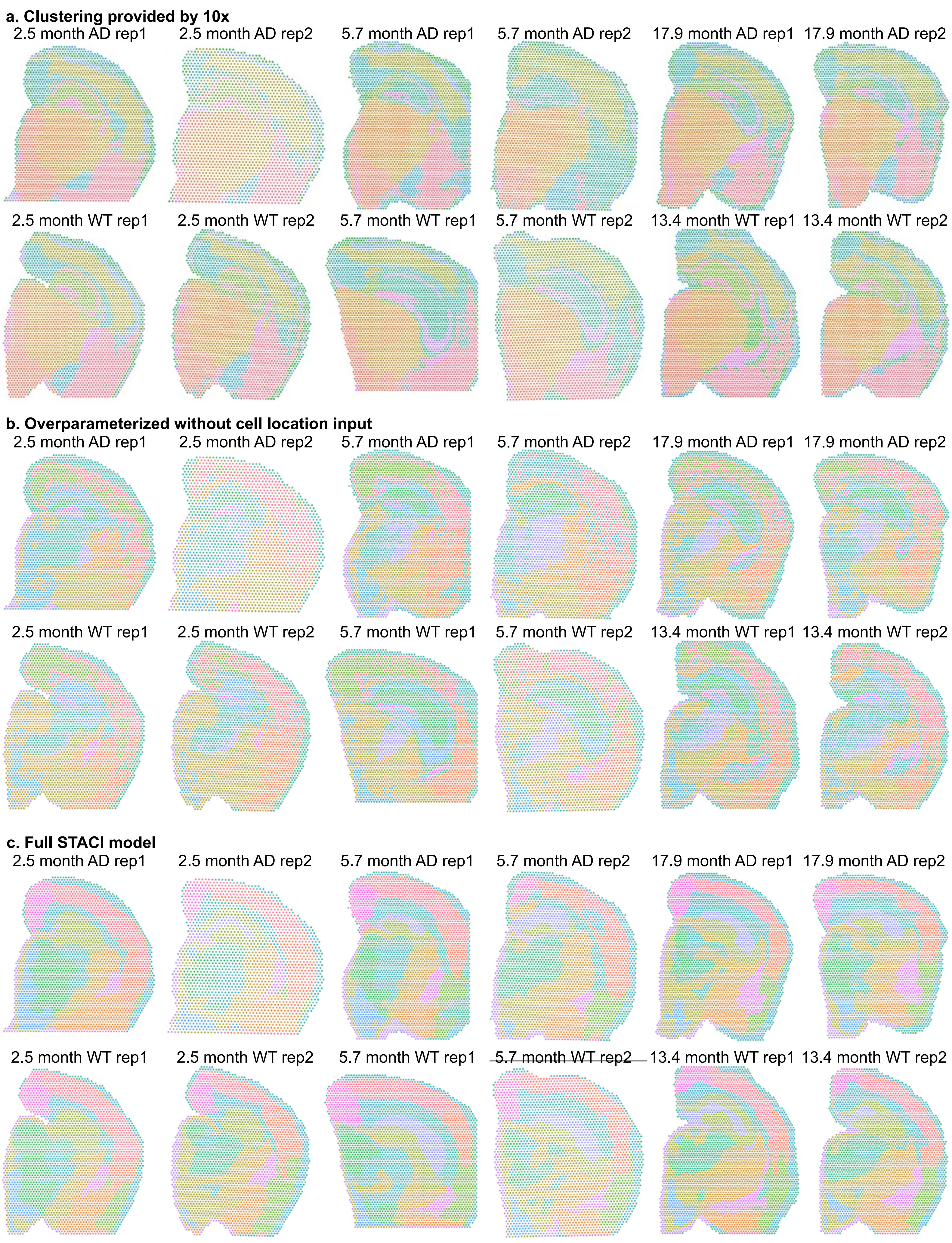

Supplementary Figure 10

Supplementary Figure 10. Validation of STACI on 10x Visium dataset of 12 mouse brain coronal sections[2].

a) Clustering of gene expression provided on the 10x website[2].
b) Leiden clustering of STACI latent space without using cell adjacency as input and with a latent dimension of 30000. The clustering resolution is 0.4.
c) Leiden clustering of the full STACI model with a latent dimension of 30000. The clustering resolution is 0.4. This clustering result shows consistent tissue segmentation across all samples and is consistent with known anatomical regions of mouse brains. In contrast, other existing methods for the analysis of spatial transcriptomics data result in having spots from different anatomical regions in the same clusters. For example, the tutorial of stLearn[3] also shows an application to 10x Visium data of mouse brain coronal sections (https://stlearn.readthedocs.io/en/latest/tutorials/stSME_clustering.html), but one cluster contains cells from the hippocampus region, isocortex, olfactory areas, and cortical subplate (cluster 18) and another cluster contains spots from both hypothalamus and cerebral nuclei (cluster 4). The Giotto package's implementation of HMRF[4] also demonstrates the results on 10x Visium data of mouse brain coronal sections (https://rubd.github.io/Giotto_site/articles/mouse_visium_brain_201226.html). While the accuracy and resolution of the spatial domains identified by HMRF and STACI are mostly comparable, some HMRF domains contain spots from different known anatomical regions of the brain in the same domains; for example, domain 16 contains cells from both inside and outside of the hypothalamus. Similarly, MUSE[5], another existing method for the analysis of spatial transcriptomics data, shows mixing of cells from different brain regions into the same cluster (see Figure 6e[5]); for example, clusters 1, 4, and 15 show mixing of cells from the isocortex (CTX), hypothalamus (HY), and olfactory area (OLF), and cluster 0 contains a mixture of cells from the hippocampus and various other regions.

Clustering provided by 10x

Cluster 5

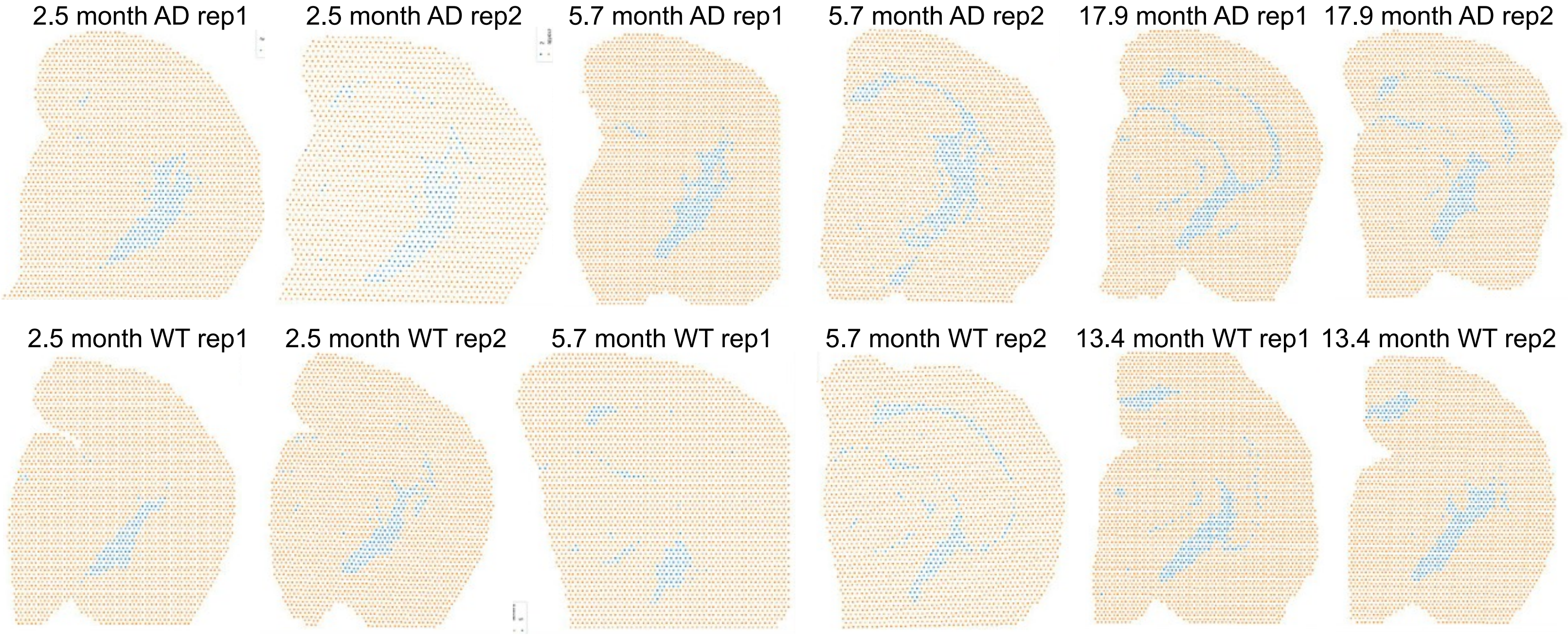

Cluster 14

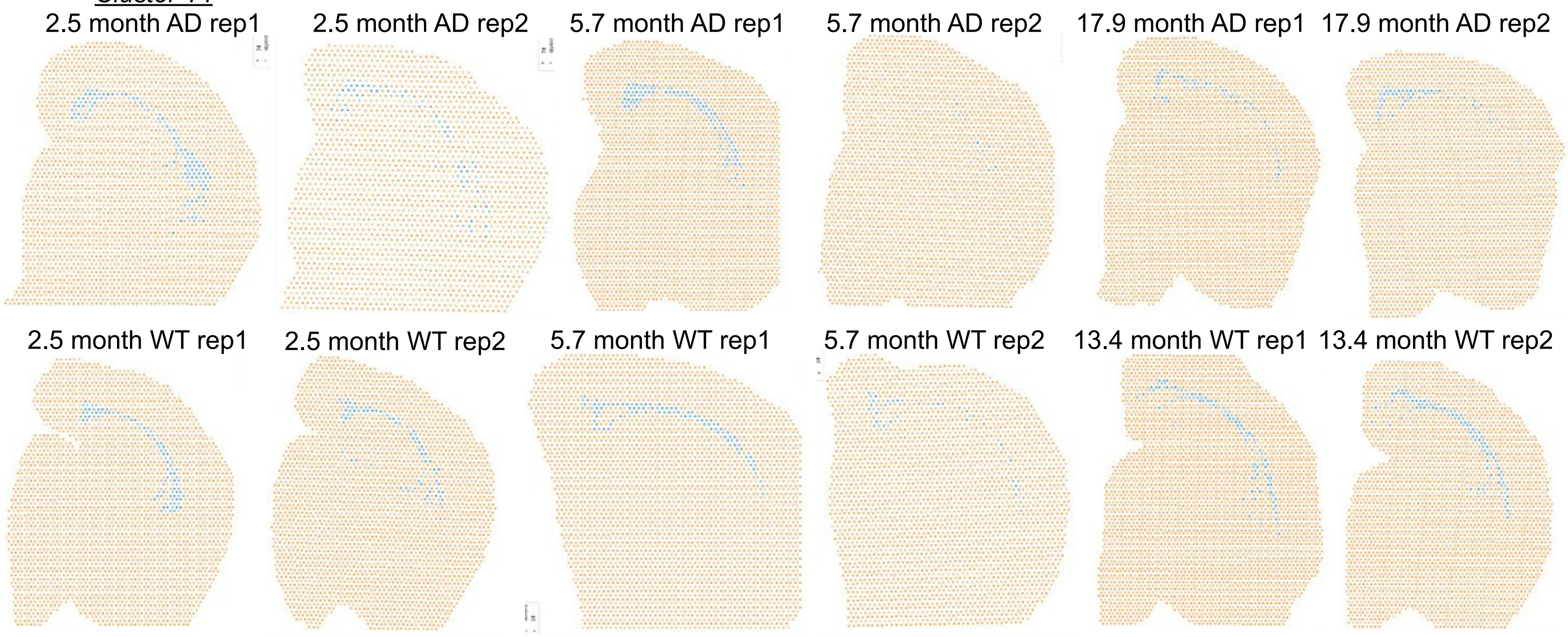

Supplementary Figure 11

Supplementary Figure 11. Segmentation of the hippocampus regions according to the clustering of gene expression provided on the 10x website[2].

Cells in cluster 5 or cluster 14 are colored blue in the respective plots.

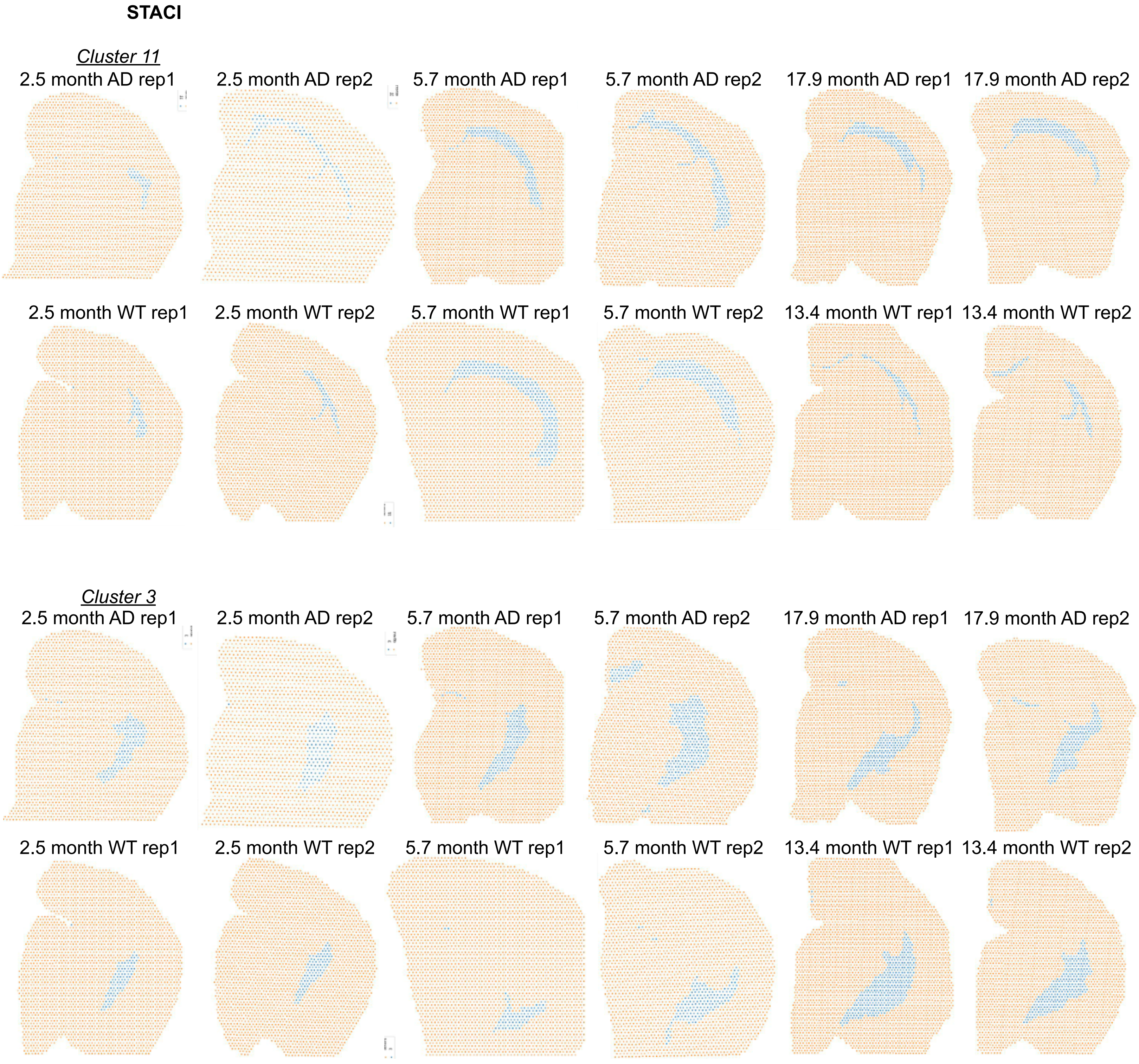

Supplementary Figure 12

Supplementary Figure 12. Segmentation of the hippocampus regions according to the clustering in the latent space of the full STACI model with a latent dimension of 30000.

Cells in cluster 11 or cluster 3 are colored blue in the respective plots.

a. Underparameterized model with standard preprocessing and batch correction

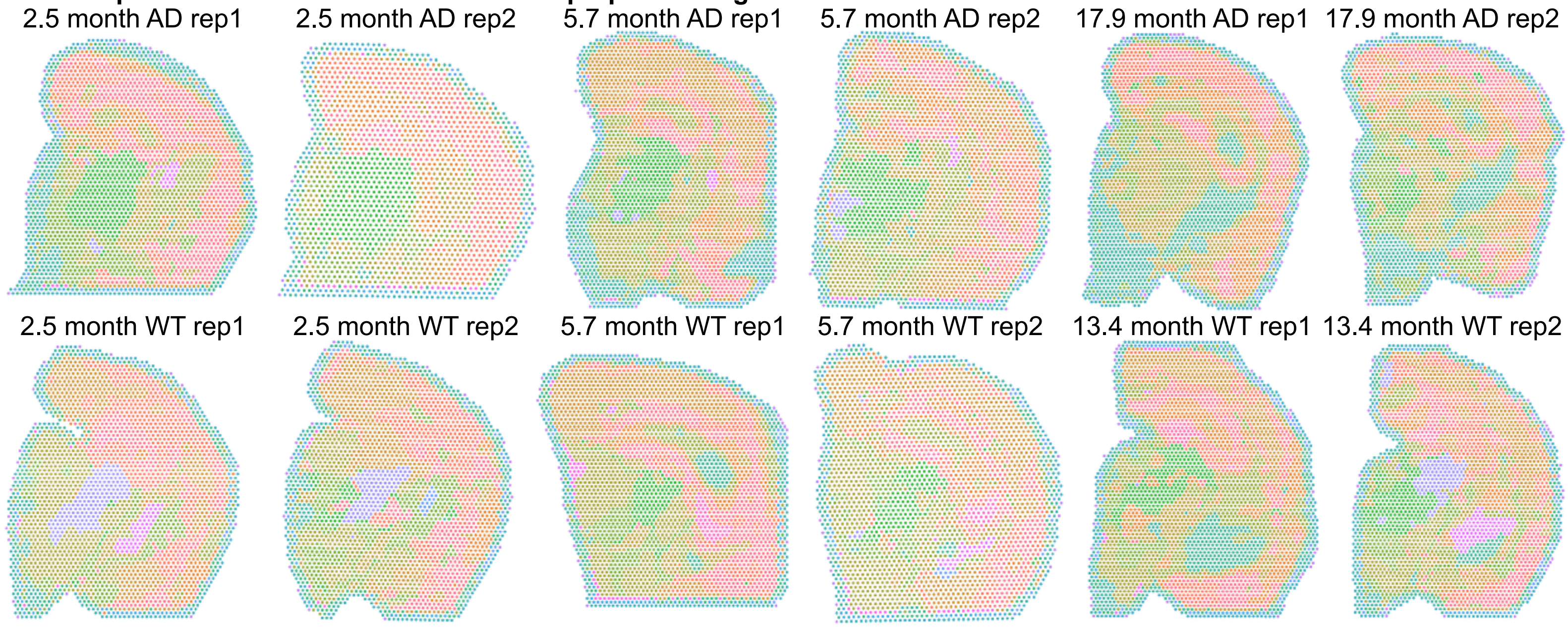

b. Cells in cluster 0 (blue) of the underparameterized model with standard processing

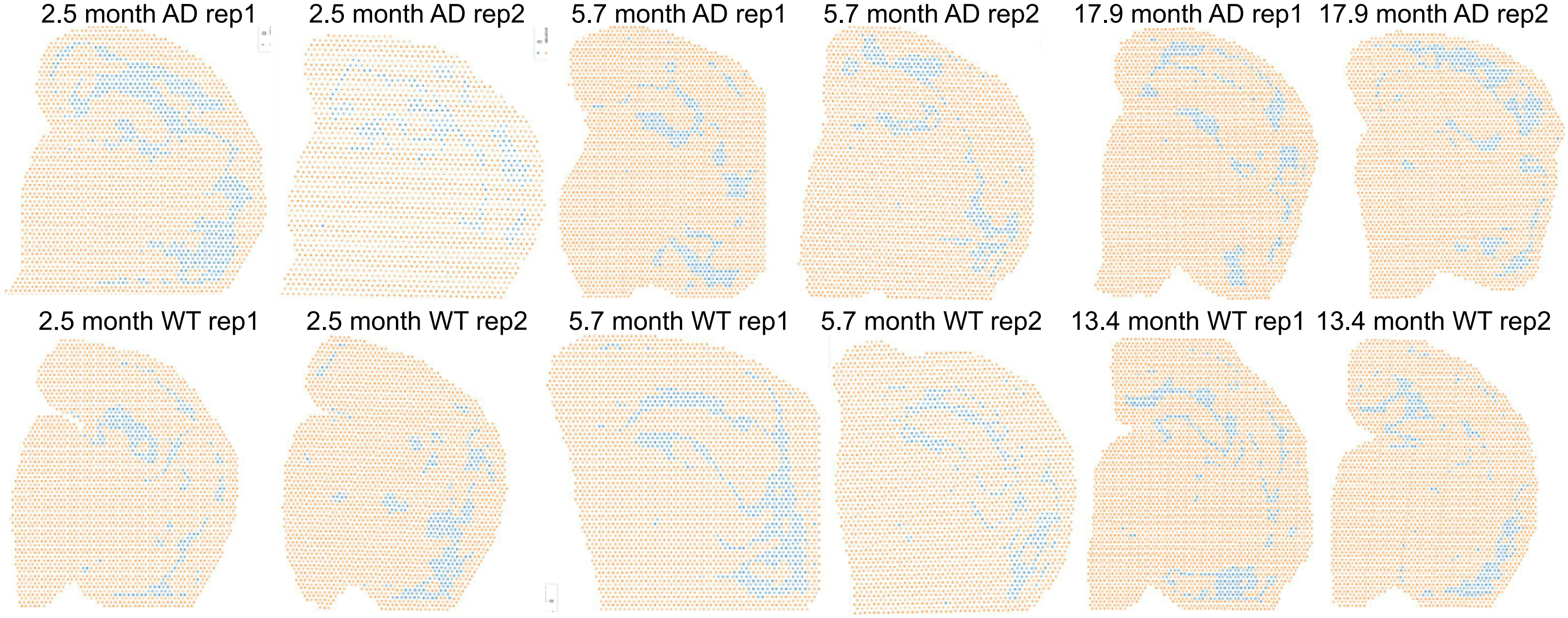

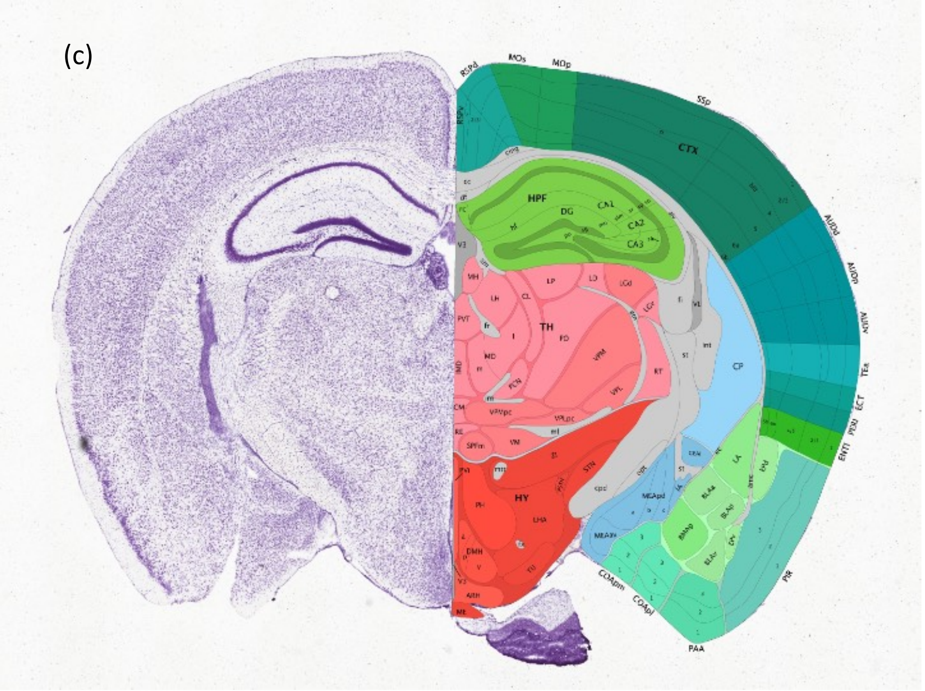

Supplementary Figure 13

Supplementary Figure 13. Application of under-parameterized STACI model on 10x Visium dataset of 12 mouse brain coronal sections[1], where the gene expression data was pre-processed by mutual nearest neighbors (MNN)[2] for batch effect removal.

a) Leiden clustering (with a resolution of 0.8) of the resulting latent space when training an under-parameterized STACI model with a latent dimension size of 32 and 17,186 input genes. Before using the pre-processed data in the autoencoder model, we applied library-size normalization, log transformation, and selected highly variable genes as the input to the MNN batch correction method as described in the MNN paper[2].
b) Cells in cluster 0 of the clustering result described in a) are highlighted in blue, all other cells are colored in orange, showing that the tissue regions included in cluster 0 are not consistent across the different samples and that cluster 0 does not correspond to a well-defined anatomical region in any of the samples.
c) A reference slice from the Allen Mouse Brain Atlas and Allen Reference Atlas - Mouse Brain[7] showing approximately the same anatomical region as in the 10x Visium data.

Name	Self CPU %	Self CPU	CPU total %	CPU total	CPU time avg	Self CUDA	Self CUDA %	CUDA total	CUDA time avg	CPU Mem	Self CPU Mem	CUDA Mem	Self CUDA Mem	# of Calls
aten::mm	0.05%	17.132 ms	37.99%	12.595 s	74.971ms	16.370s	64.57%	16.648s	99.094ms	8 b	8 b	160.44 Gb	136.66 Gb	168
void cutlass::Kernel<cutlass_80_tensorop_s1688gemm_1...	0.00%	0.000us	0.00%	0.000us	0.000us	4.312s	17.01%	4.312s	165.862 ms	0 b	0 b	0 b	0 b	26
void cutlass::Kernel<cutlass_80_tensorop_s1688gemm_1...	0.00%	0.000us	0.00%	0.000us	0.000us	4.296s	16.95%	4.296s	143.210 ms	0 b	0 b	0 b	0 b	30
aten::addmm	0.05%	15.090 ms	53.30%	17.668 s	196.310 ms	4.085s	16.11%	4.443s	49.364ms	0 b	0 b	42.00 Gb	-9.95 Gb	90
void cutlass::Kernel<cutlass_80_tensorop_s1688gemm_1...	0.00%	0.000us	0.00%	0.000us	0.000us	3.606s	14.23%	3.606s	78.402ms	0 b	0 b	0 b	0 b	46
aten::copy_	0.15%	48.478 ms	75.19%	24.925 s	27.151ms	3.202s	12.63%	3.202s	3.488ms	8 b	32 b	3.70 Gb	875.33 Mb	918
void cutlass::Kernel<cutlass_80_tensorop_s1688gemm_1...	0.00%	0.000us	0.00%	0.000us	0.000us	2.780s	10.96%	2.780s	79.419ms	0 b	0 b	0 b	0 b	35
Memcpy PtoP (Device -> Device)	0.00%	0.000us	0.00%	0.000us	0.000us	2.293s	9.05%	2.293s	8.494ms	0 b	0 b	0 b	0 b	270
void cutlass::Kernel<cutlass_80_tensorop_s1688gemm_1...	0.00%	0.000us	0.00%	0.000us	0.000us	2.219s	8.75%	2.219s	138.662 ms	0 b	0 b	0 b	0 b	16
void cutlass::Kernel<cutlass_80_tensorop_s1688gemm_2...	0.00%	0.000us	0.00%	0.000us	0.000us	1.959s	7.73%	1.959s	78.340ms	0 b	0 b	0 b	0 b	25
	**Self CPU time total: 33.150s**													
	**Self CUDA time total: 25.352s**													

Name	Self CPU %	Self CPU	CPU total %	CPU total	CPU time avg	Self CUDA	Self CUDA %	CUDA total	CUDA time avg	CPU Mem	Self CPU Mem	CUDA Mem	Self CUDA Mem	# of Calls
aten::mm	0.08%	22.054 ms	37.22%	10.235 s	60.923ms	13.372s	63.20%	13.600s	80.952ms	0 b	0 b	153.07 Gb	130.38 Gb	168
void cutlass::Kernel<cutlass_80_tensorop_s1688gemm_1...	0.00%	0.000us	0.00%	0.000us	0.000us	3.564s	16.84%	3.564s	118.791 ms	0 b	0 b	0 b	0 b	30
aten::addmm	0.07%	20.012 ms	52.46%	14.427 s	160.300 ms	3.443s	16.27%	3.736s	41.510ms	0 b	-8 b	33.62 Gb	-5.94 Gb	90
void cutlass::Kernel<cutlass_80_tensorop_s1688gemm_1...	0.00%	0.000us	0.00%	0.000us	0.000us	3.207s	15.16%	3.207s	89.079ms	0 b	0 b	0 b	0 b	36
void cutlass::Kernel<cutlass_80_tensorop_s1688gemm_1...	0.00%	0.000us	0.00%	0.000us	0.000us	2.897s	13.69%	2.897s	80.469ms	0 b	0 b	0 b	0 b	36
aten::copy_	0.20%	56.220 ms	74.44%	20.472 s	22.301ms	2.874s	13.58%	2.874s	3.131ms	16 b	8 b	7.20 Gb	7.24 Gb	918
void cutlass::Kernel<cutlass_80_tensorop_s1688gemm_1...	0.00%	0.000us	0.00%	0.000us	0.000us	2.205s	10.42%	2.205s	73.510ms	0 b	0 b	0 b	0 b	30
Memcpy PtoP (Device -> Device)	0.00%	0.000us	0.00%	0.000us	0.000us	1.877s	8.87%	1.877s	6.953ms	0 b	0 b	0 b	0 b	270
void cutlass::Kernel<cutlass_80_tensorop_s1688gemm_2...	0.00%	0.000us	0.00%	0.000us	0.000us	1.689s	7.98%	1.689s	112.608 ms	0 b	0 b	0 b	0 b	15
void cutlass::Kernel<cutlass_80_tensorop_s1688gemm_1...	0.00%	0.000us	0.00%	0.000us	0.000us	1.677s	7.93%	1.677s	83.858ms	0 b	0 b	0 b	0 b	20
	**Self CPU time total: 27.501s**													
	**Self CUDA time total: 21.160s**													

Supplementary Figure 14

Supplementary Figure 14. The CPU and GPU usage of training STACI for 6 epochs based on the 12 10x Visium samples of mouse brain coronal sections.

Two different usage samples at different epoch numbers are provided. The table lists the top ten functions by the total CUDA time ("self_cuda_time_total").

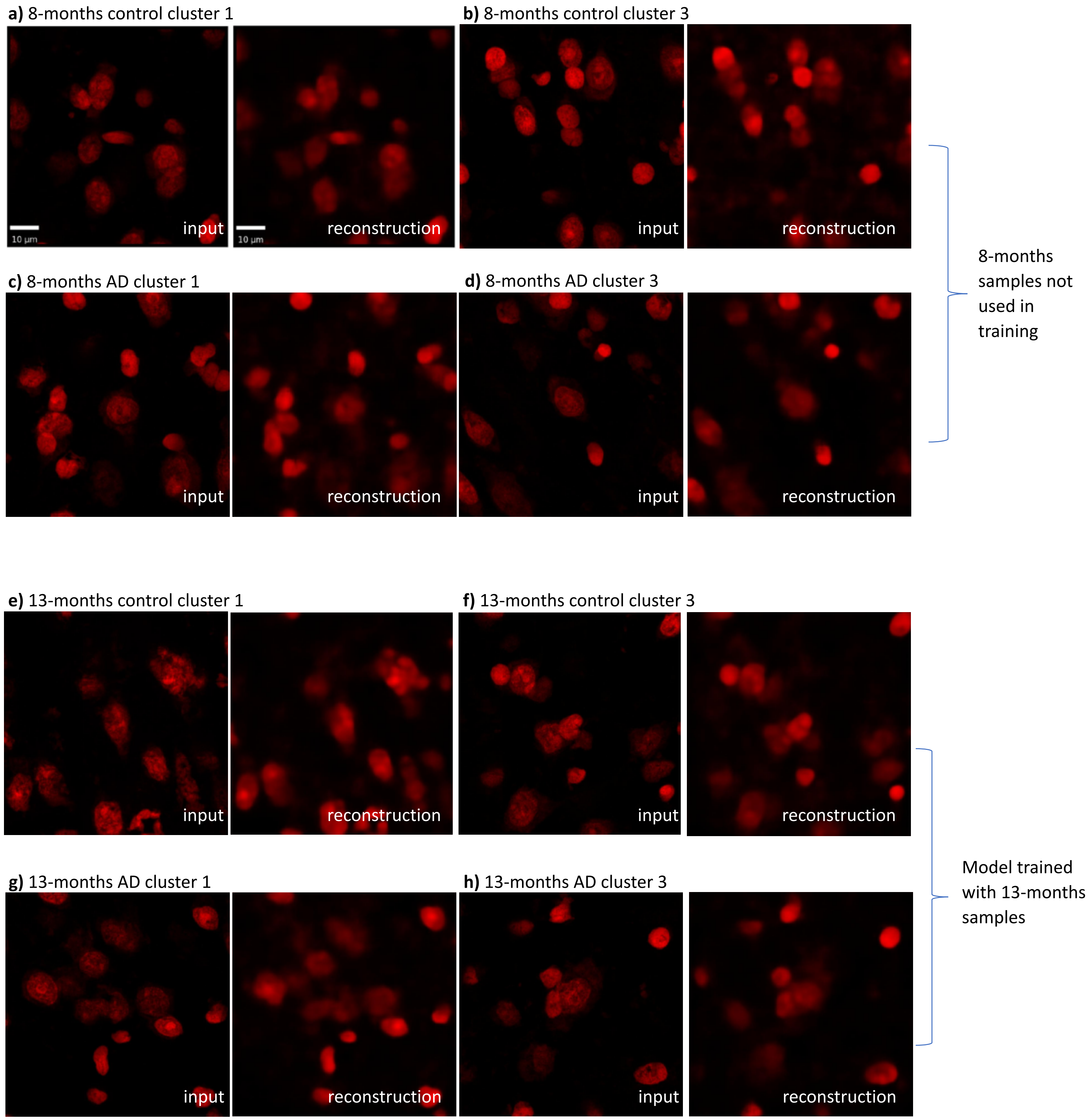

Supplementary Figure 15

Supplementary Figure 15. Examples of input chromatin images to the CNN autoencoder and the corresponding reconstructed images. This is the result of the STACI model with over-parameterization and latent space matching of gene expression, cell adjacencies, and chromatin images (latent size = 5000). Only the 13-month samples were used in training of both, the graph autoencoder for gene expression and cell adjacencies as well as the CNN autoencoder for chromatin images. Each panel contains one representative example of an input chromatin image and the corresponding reconstruction from one of the four mice samples as listed below. Each mouse sample contains at least 7257 cells.
a) 8-months control cluster 1; b) 8-months control cluster 3; c) 8-months AD cluster 1; d) 8-months AD cluster 3; e) 13-months control cluster 1; f) 13-months control cluster 3; g) 13-months AD cluster 1; h) 13-months AD cluster 3.

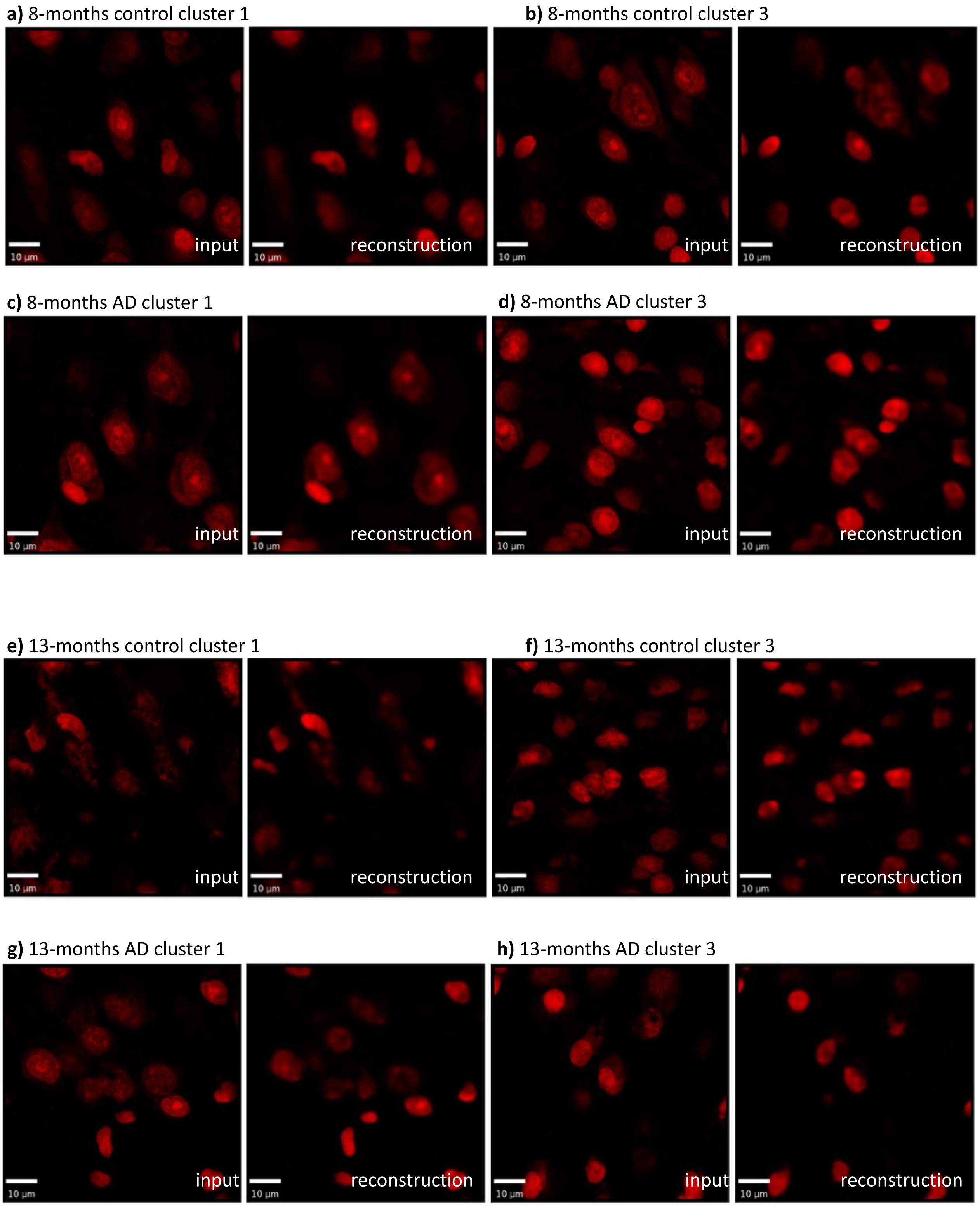

Supplementary Figure 16

Supplementary Figure 16. Examples of input chromatin images to the CNN autoencoder and the corresponding reconstructed images. There is no latent space matching with the graph autoencoder. All samples were used in training the CNN autoencoder. Each panel contains one representative example of an input chromatin image and the corresponding reconstruction from one of the four mice samples as listed below. Each mouse sample contains at least 7257 cells.
a) 8-months control cluster 1; b) 8-months control cluster 3; c) 8-months AD cluster 1; d) 8-months AD cluster 3; e) 13-months control cluster 1; f) 13-months control cluster 3; g) 13-months AD cluster 1; h) 13-months AD cluster 3.

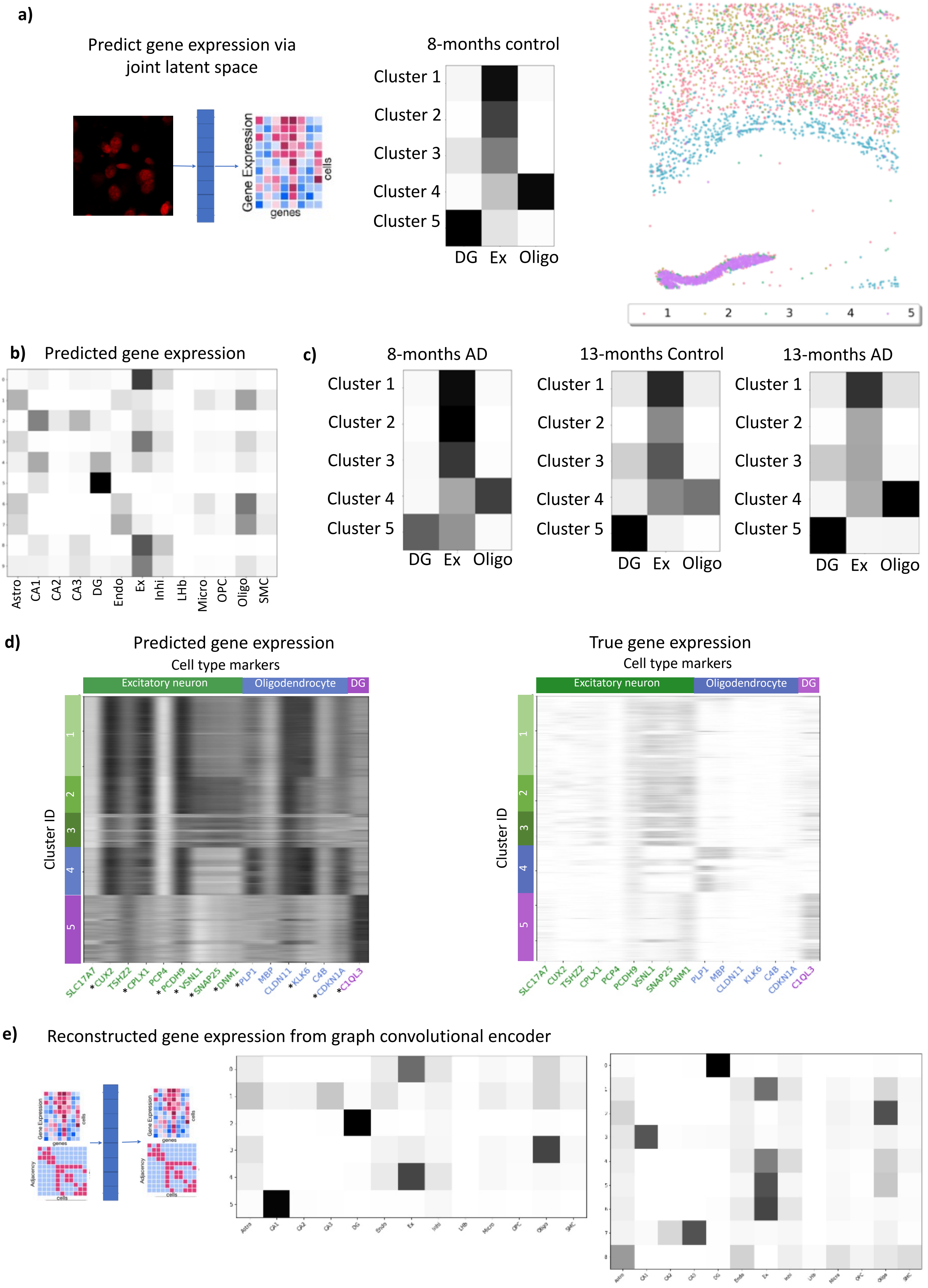

Supplementary Figure 17

Supplementary Figure 17. Joint latent space allows for the translation of chromatin images of a new sample to gene expression.

a) Cell type composition in each cluster of the predicted gene expression in the 8-months control sample as annotated by Zeng *et al*.[1] (middle). The clusters of predicted gene expression profiles were plotted by the cell locations in the 8-months control sample (right). Excitatory neurons (excluding CA1, CA2, CA3), DG, and oligodendrocytes in the corpus callosum are included in the clustering to represent major neuronal and glial cell types. All tissue samples were used in the clustering.
b) Cell type composition in each cluster of the predicted gene expression profiles in the 8-months control sample as annotated by Zeng *et al*.[1]. All cells in all tissue samples were used in the clustering.
c) Cell type composition in each cluster of the predicted gene expression profiles as annotated by Zeng *et al.*[1]. Excitatory neurons (excluding CA1, CA2, CA3), dentate gyrus (DG), and oligodendrocytes (Oligo) in the corpus callosum are included in the clustering to represent major neuronal and glial cell types. This is the same clustering as a).
d) Top: Predicted expression of known cell type markers in each cluster of predicted gene expression profiles. This is the same clustering as a). All samples are included in the plot. *: differentially expressed cluster markers for each cell type; p-val < 0.05 and fold change > 1.1. Bottom: True gene expression of known cell type markers in each cluster of predicted gene expression profiles. All samples are included in the plot.

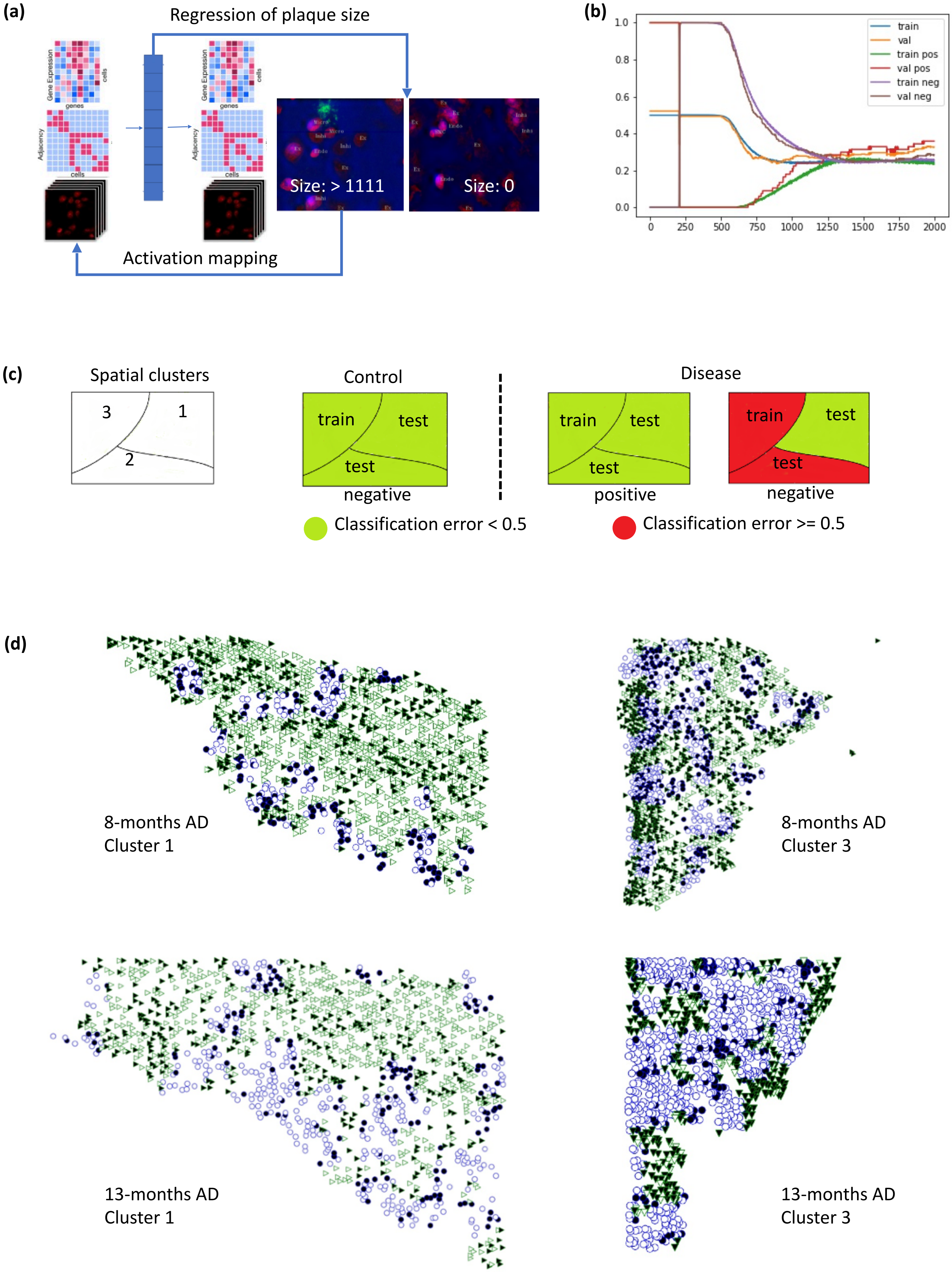

Supplementary Figure 18

Supplementary Figure 18. Regression analysis of the joint latent representation predicts plaque size near cells and identifies disease progression across the cortex.

a) The joint latent representation of gene expression, cell adjacencies, and chromatin images are used as the input to a regression model to predict the presence and size of nearby plaque. Plaque images are preprocessed with a size threshold of 1111 pixels. The gradient of the regression output was calculated with respect to the input chromatin images.
b) Training and validation accuracies of the regression model trained with cluster 3 in the 13-months control and AD samples (see Supplementary Table, model #18).
c) Training and test classification errors in the cortex regions of 13-months samples. The regression model trained on cluster 3 can be generalized to cluster 1 but cannot be overfitted to distinguish positive and negative samples in cluster 3 (see Supplementary Table, model #18).
d) Positive (blue circle) and negative (green triangle) cells plotted at their locations in tissue samples. Black means wrong classification. White means correct classification.

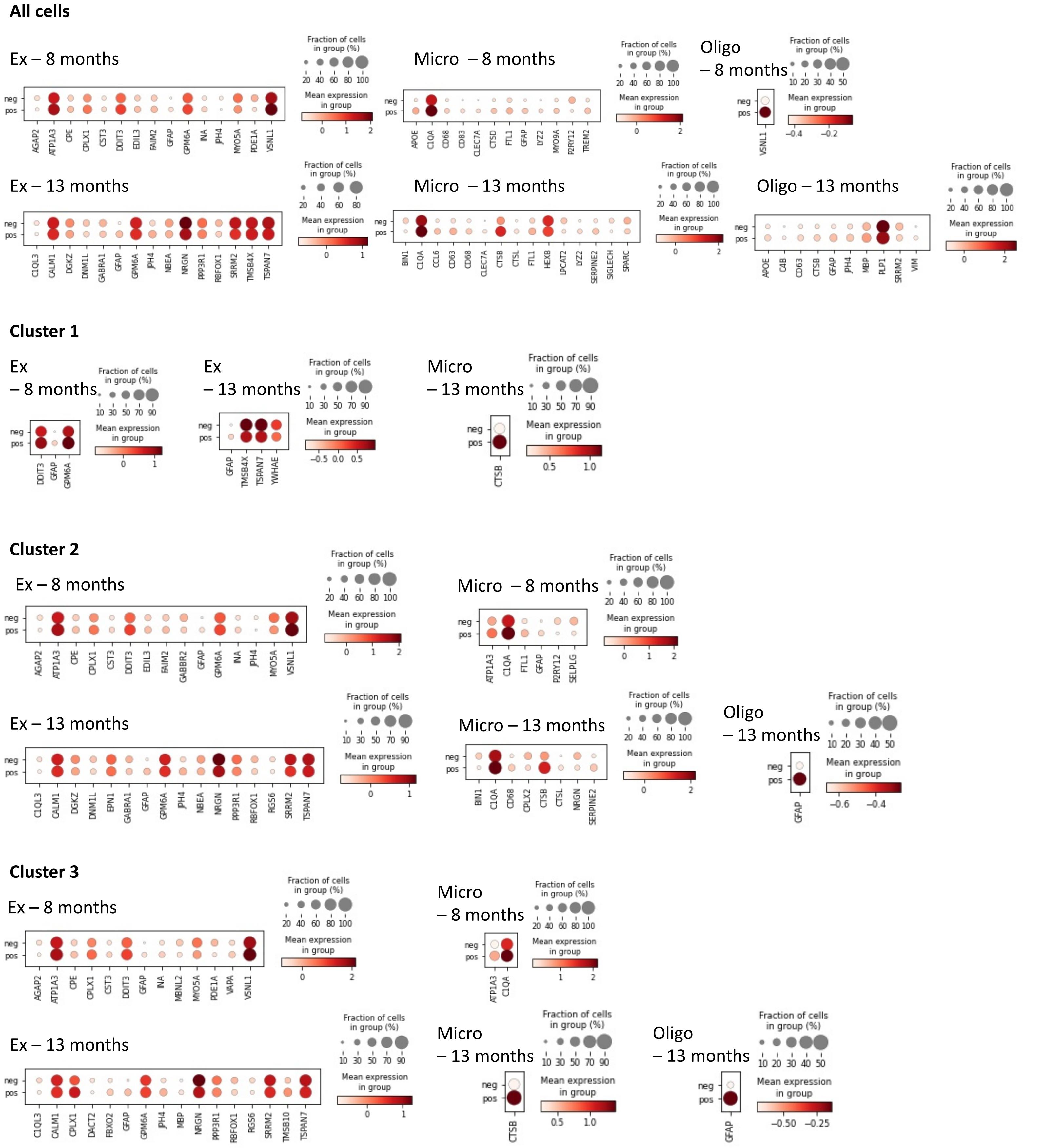

Supplementary Figure 19

Supplementary Figure 19. Differential expression analysis of cells close to and far from plaques. Positive cells (pos) close to plaques and negative cells (neg) far from plaques are defined as in the regression model. A cell is positive if there is plaque within the 75.68 µm x 75.68 µm image patch centered at the cell. All cells in the control samples are used as negative cells at the two time points. All cells: cells from all three cortex clusters (1, 2, 3) are combined for DE analysis. Statistical significance was defined as p-value < 0.05 after correction by Benjamini-Hochberg procedure[8] and fold change of at least 10% in either direction. At most 25 up-regulated and 25 down-regulated genes with smallest p-values are shown. Ex: excitatory neuron; Micro: microglia; Oligo: oligodendrocyte.

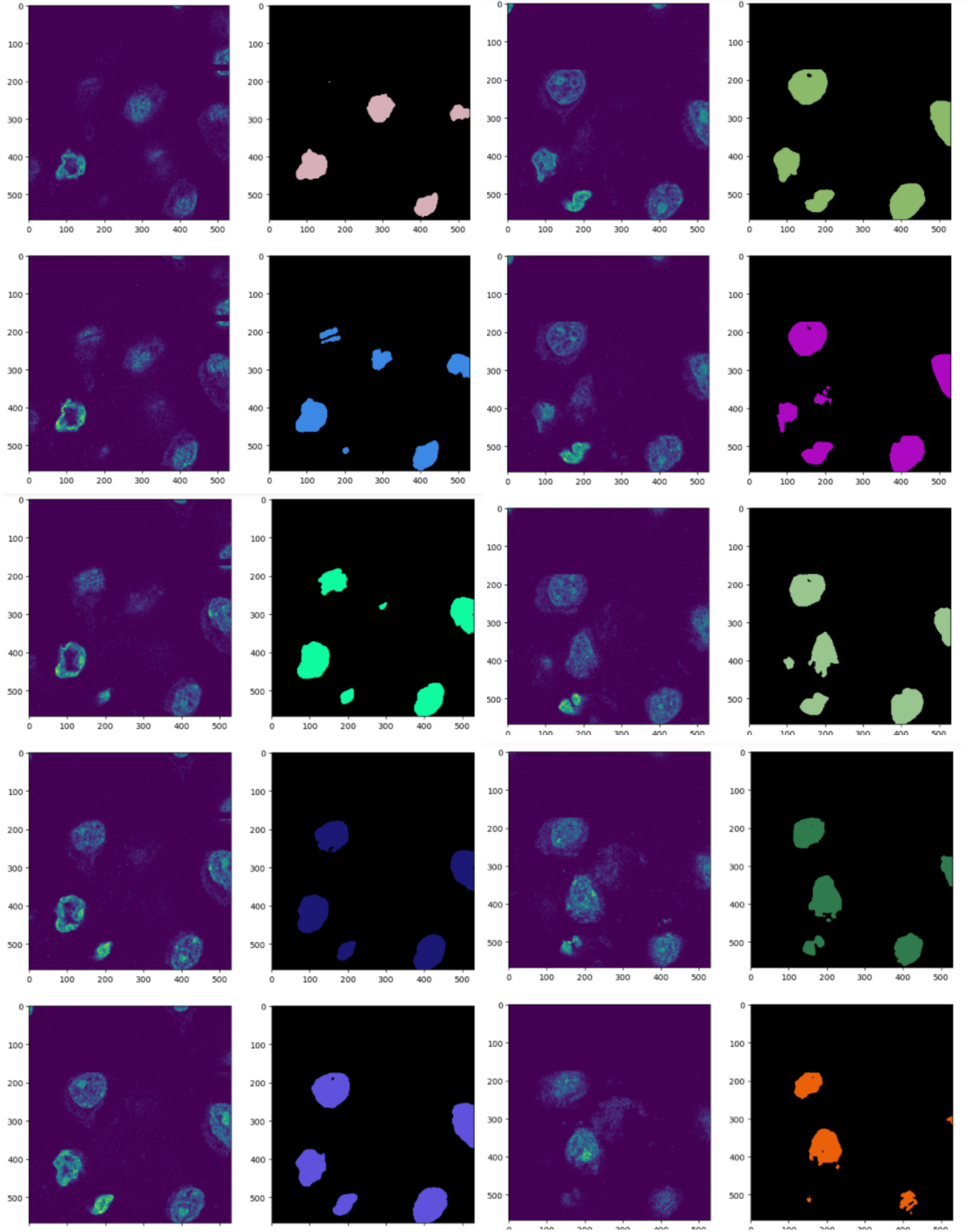

Supplementary Figure 20

Supplementary Figure 20. Example of 3D segmentation of some z positions at the same x-y location.

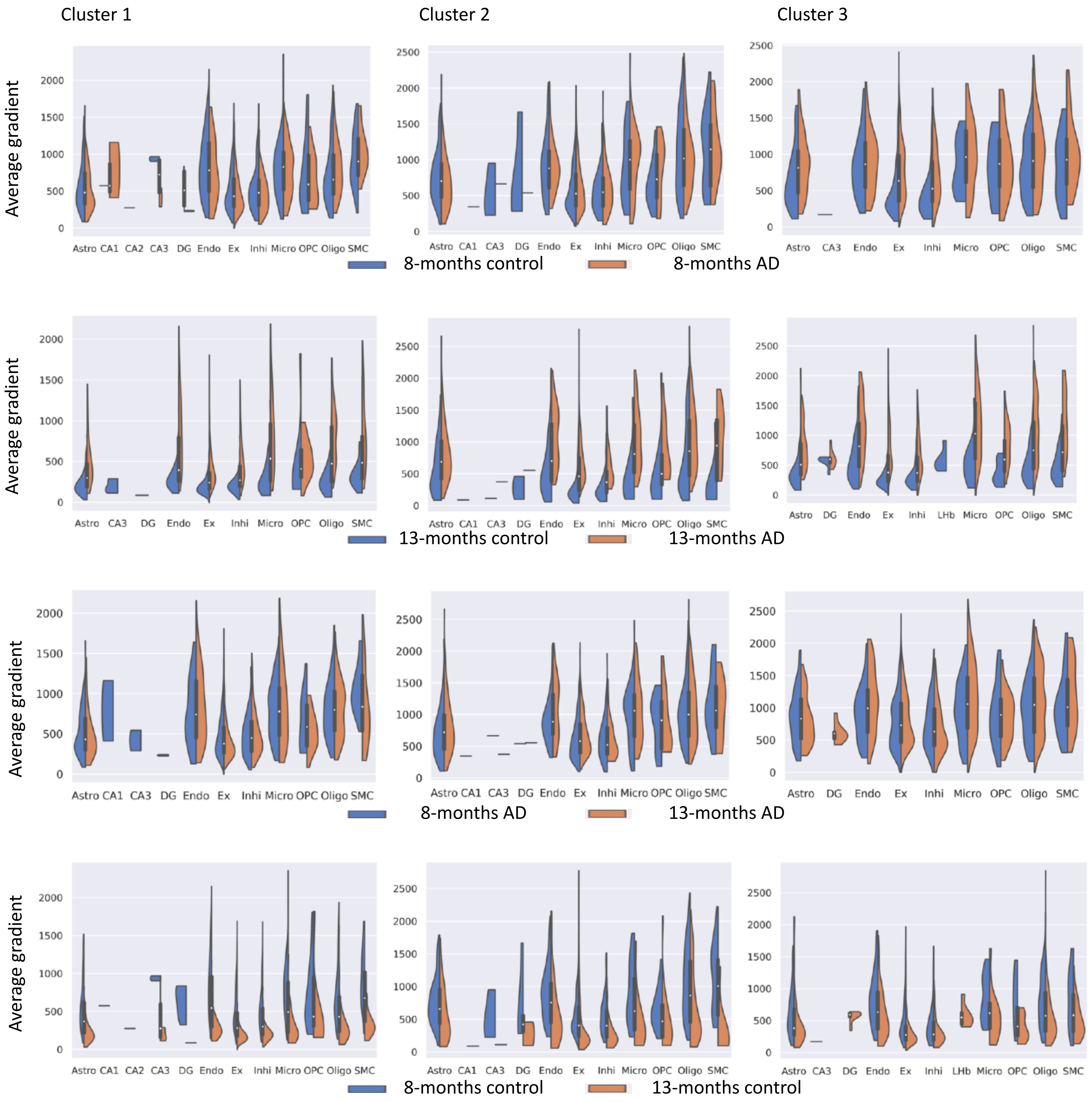

Supplementary Figure 21

Supplementary Figure 21. Average regression gradient of each cell type in different cortex clusters.

Each plot contains cells from two mice with at least 7257 cells each. Bounds of boxes in the boxplots indicate quartiles. Whiskers indicate minima and maxima. Cell types are labeled as Ex: excitatory neuron; Micro: microglia; Inhi: inhibitory neuron; Endo: endothelial cell; Astro: astrocytes; OPC: oligodendrocyte precursor cell; SMC: smooth muscle cell.

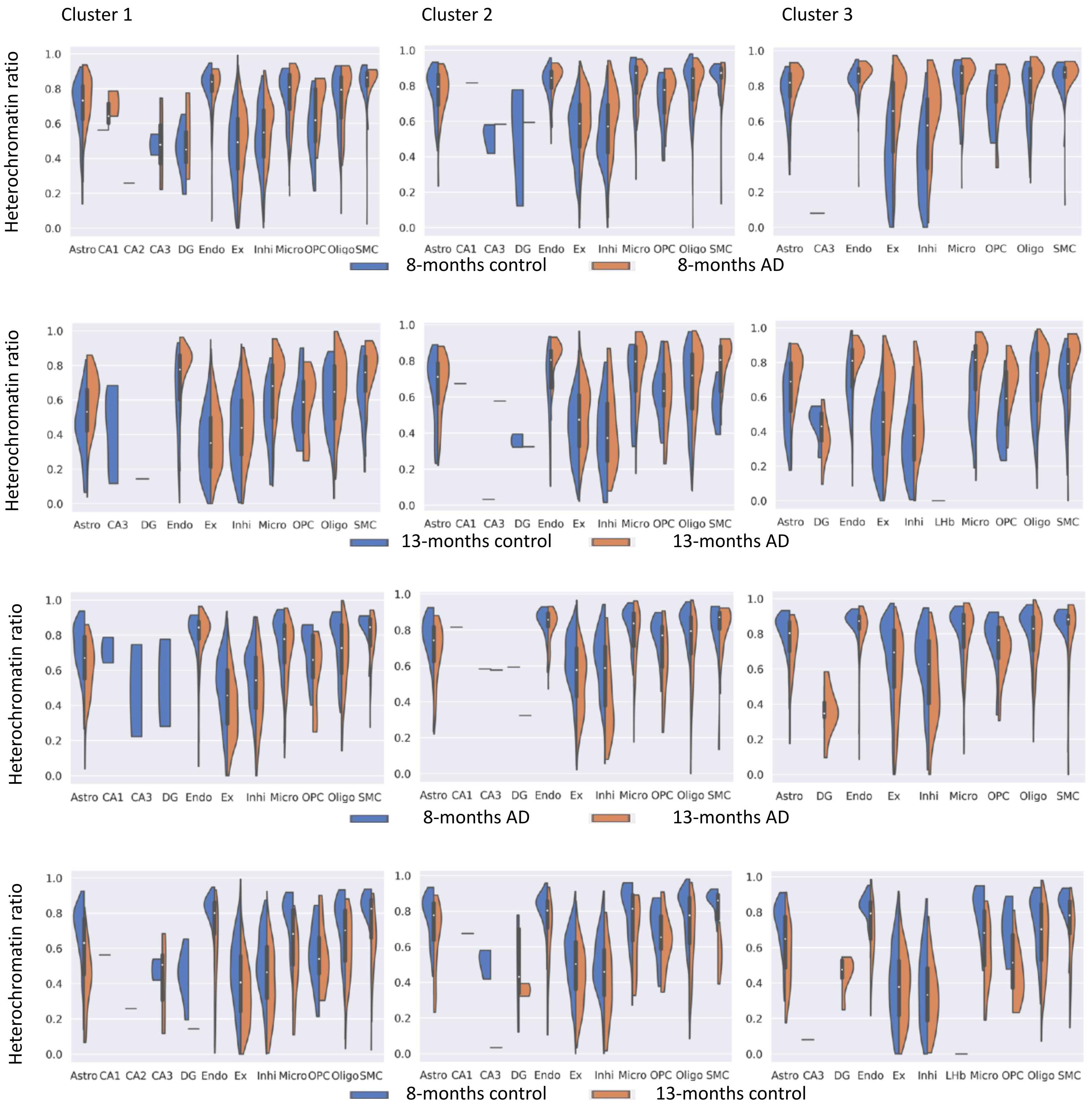

Supplementary Figure 22

Supplementary Figure 22. Heterochromatin ratio of each cell type in different cortex clusters.

Each plot contains cells from two mice with at least 7257 cells each. Bounds of boxes in the boxplots indicate quartiles. Whiskers indicate minima and maxima. Cell types are labeled as Ex: excitatory neuron; Micro: microglia; Inhi: inhibitory neuron; Endo: endothelial cell; Astro: astrocytes; OPC: oligodendrocyte precursor cell; SMC: smooth muscle cell.

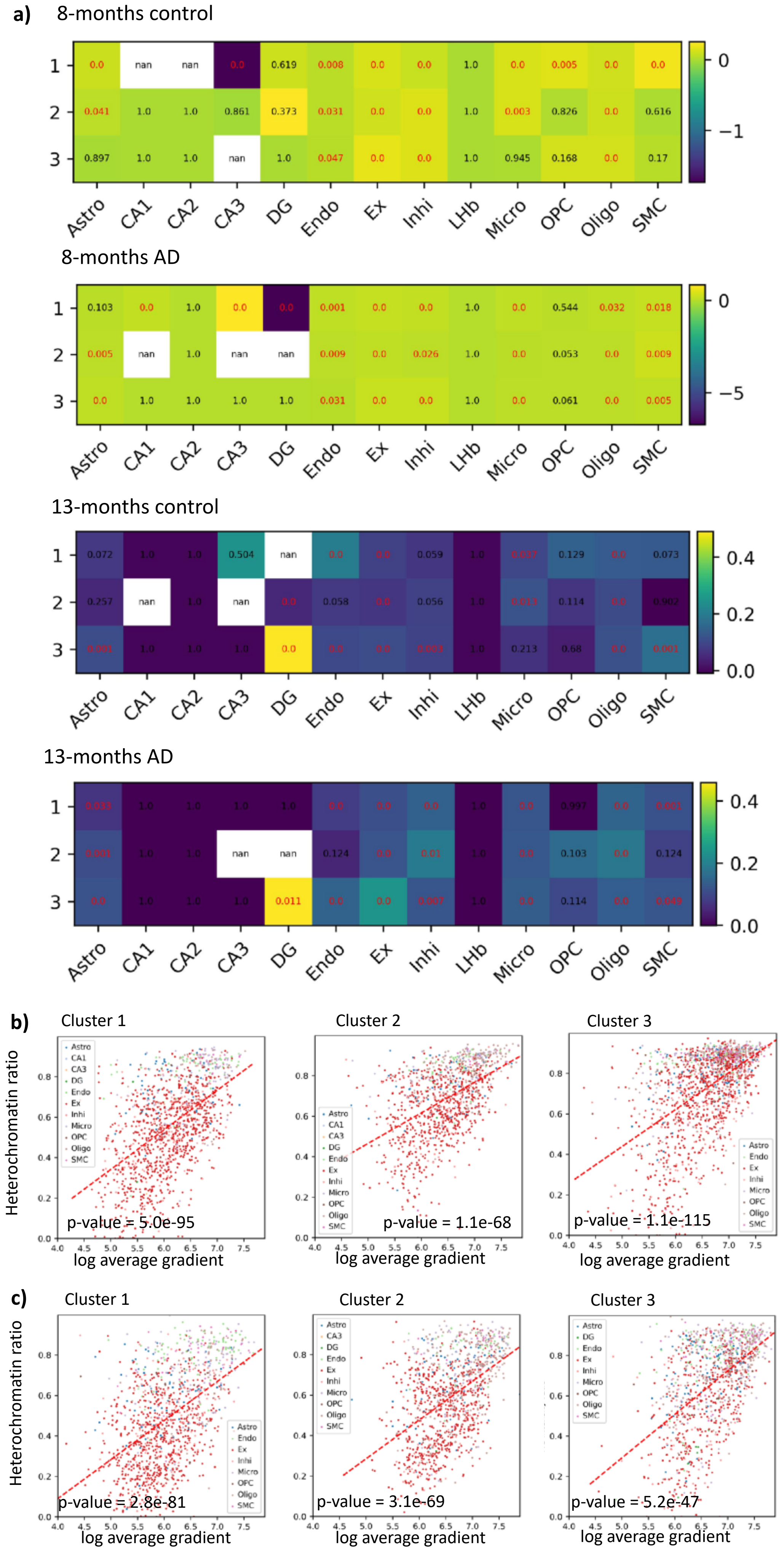

Supplementary Figure 23

Supplementary Figure 23. Linear regression of heterochromatin ratio with respect to log2 average gradient.

a) Heatmaps colored by the slope of linear regression of heterochromatin ratio vs log2 average gradient of each cell type in each cortex cluster. Numbers are p-values of the regression analysis.

b) Heterochromatin ratio vs $\log_2$ average gradient of cells in the cortex clusters of the 8-months AD sample. Red line fitted with linear regression analysis. P-values are calculated by two-sided Wald Test and the null hypothesis that the slope is 0.

c) Heterochromatin ratio vs $\log_2$ average gradient of cells in the cortex clusters of the 13-months AD sample. Red line fitted with linear regression analysis. P-values are calculated by two-sided Wald Test and the null hypothesis that the slope is 0.

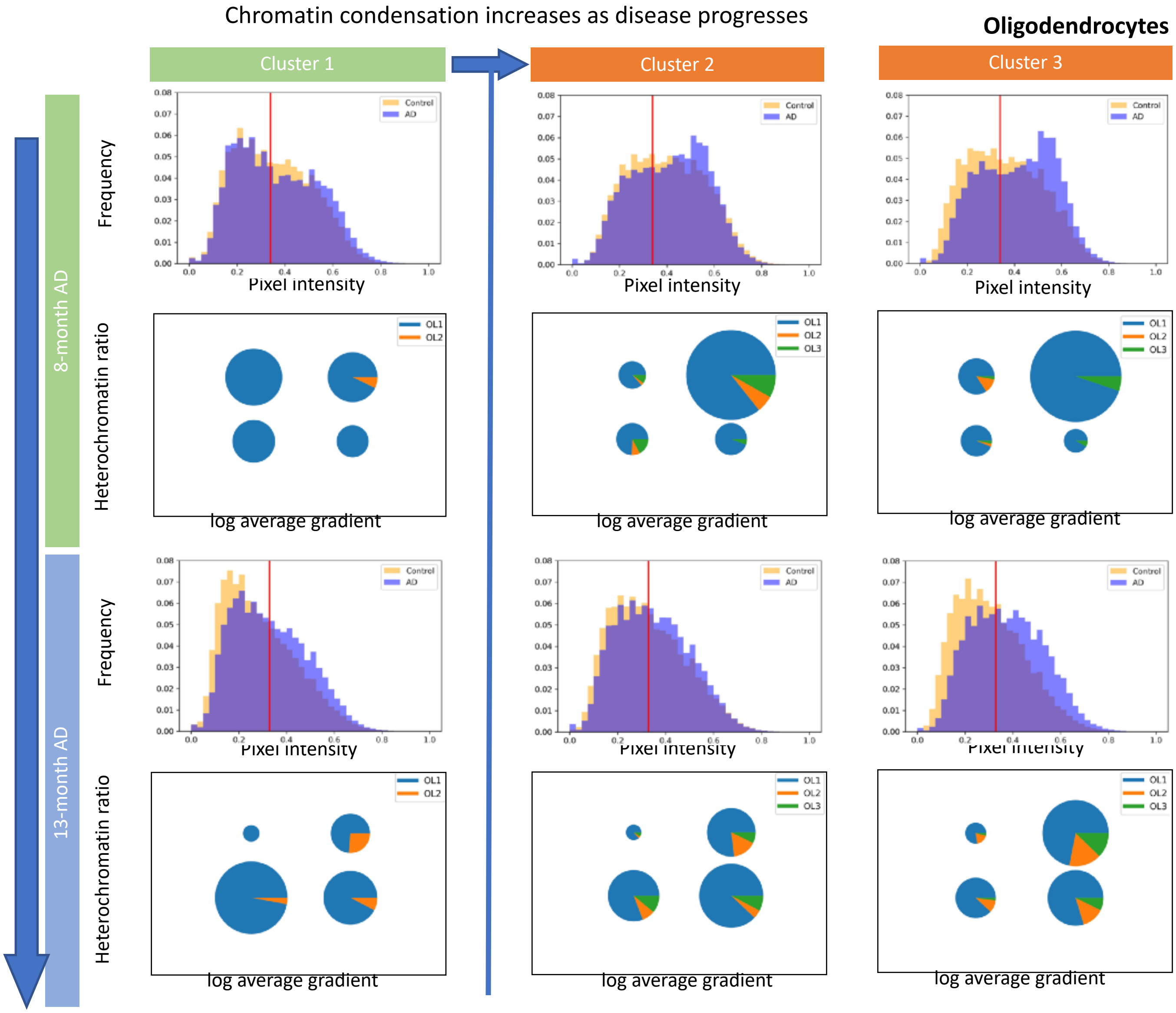

Supplementary Figure 24

Supplementary Figure 24. Chromatin condensation is indicative of the impacts of amyloid plaque on cells.

All plots are for oligodendrocytes in the different clusters at the two different time points.

Row 1 and 3: histograms of pixel intensities in all oligodendrocytes of the specific cortex cluster at each time point, normalized to sum to 1. Red line: lower threshold of heterochromatin pixels (see Methods). Pie charts: cells grouped into four quadrants by heterochromatin ratio (threshold at 0.8) and log average gradient (threshold at 6.7). The sizes of the pie charts are proportional to the fraction of cells in each quadrant with respect to all four quadrants in the same clusters. The angles in the pie charts are proportional to the fraction of each subtype in each quadrant.

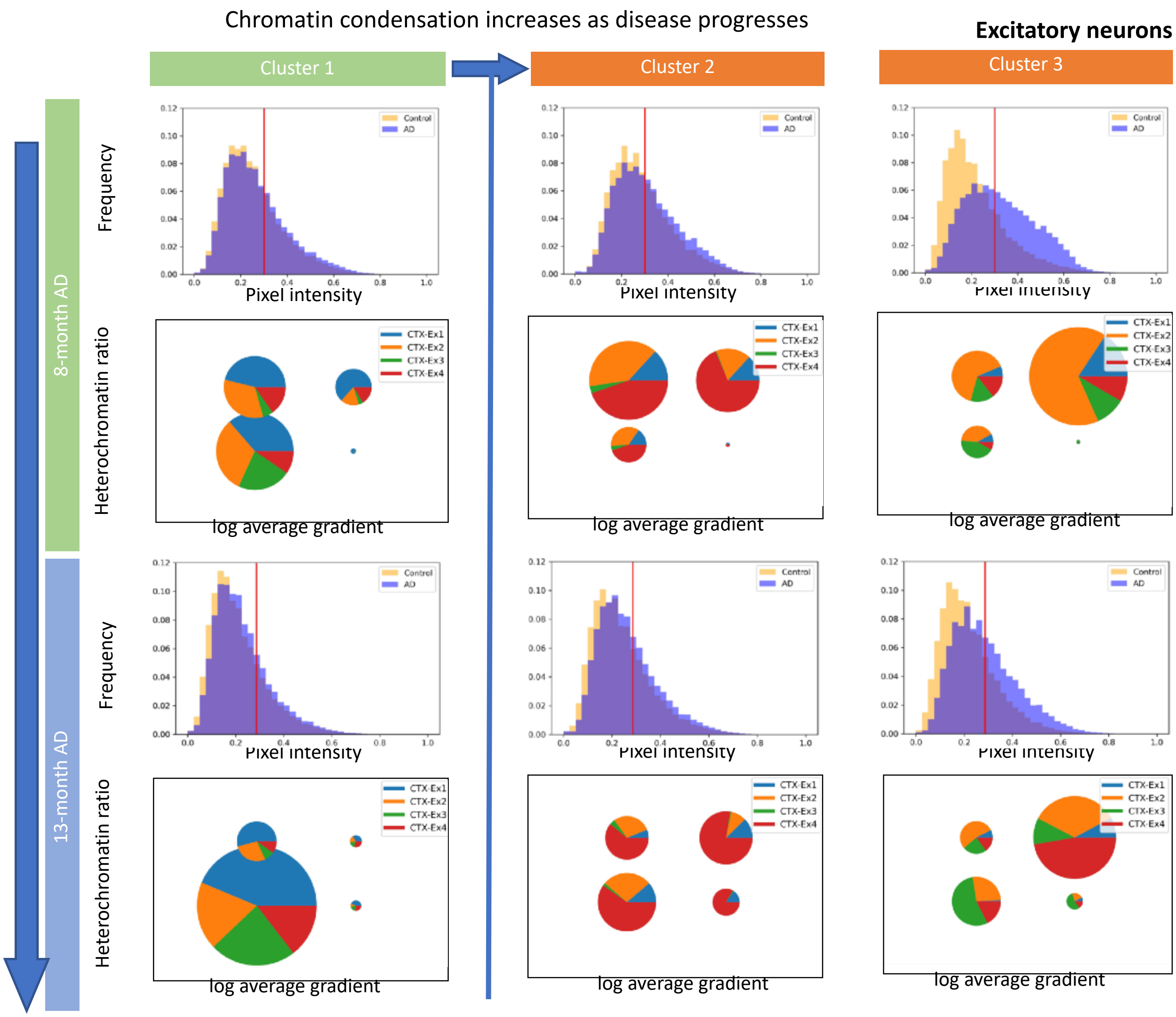

Supplementary Figure 25

Supplementary Figure 25. Chromatin condensation is indicative of the impacts of amyloid plaque on cells.

All plots are for excitatory neurons in the different clusters at the two different time points.

Row 1 and 3: histograms of pixel intensities in all excitatory neurons of the specific cortex cluster at each time point, normalized to sum to 1. Red line: lower threshold of heterochromatin pixels (see Methods). Pie charts: cells grouped into four quadrants by heterochromatin ratio (threshold at 0.5) and log average gradient (threshold at 6.5). The sizes of the pie charts are proportional to the fraction of cells in each quadrant with respect to all four quadrants in the same clusters. The angles in the pie charts are proportional to the fraction of each subtype in each quadrant.

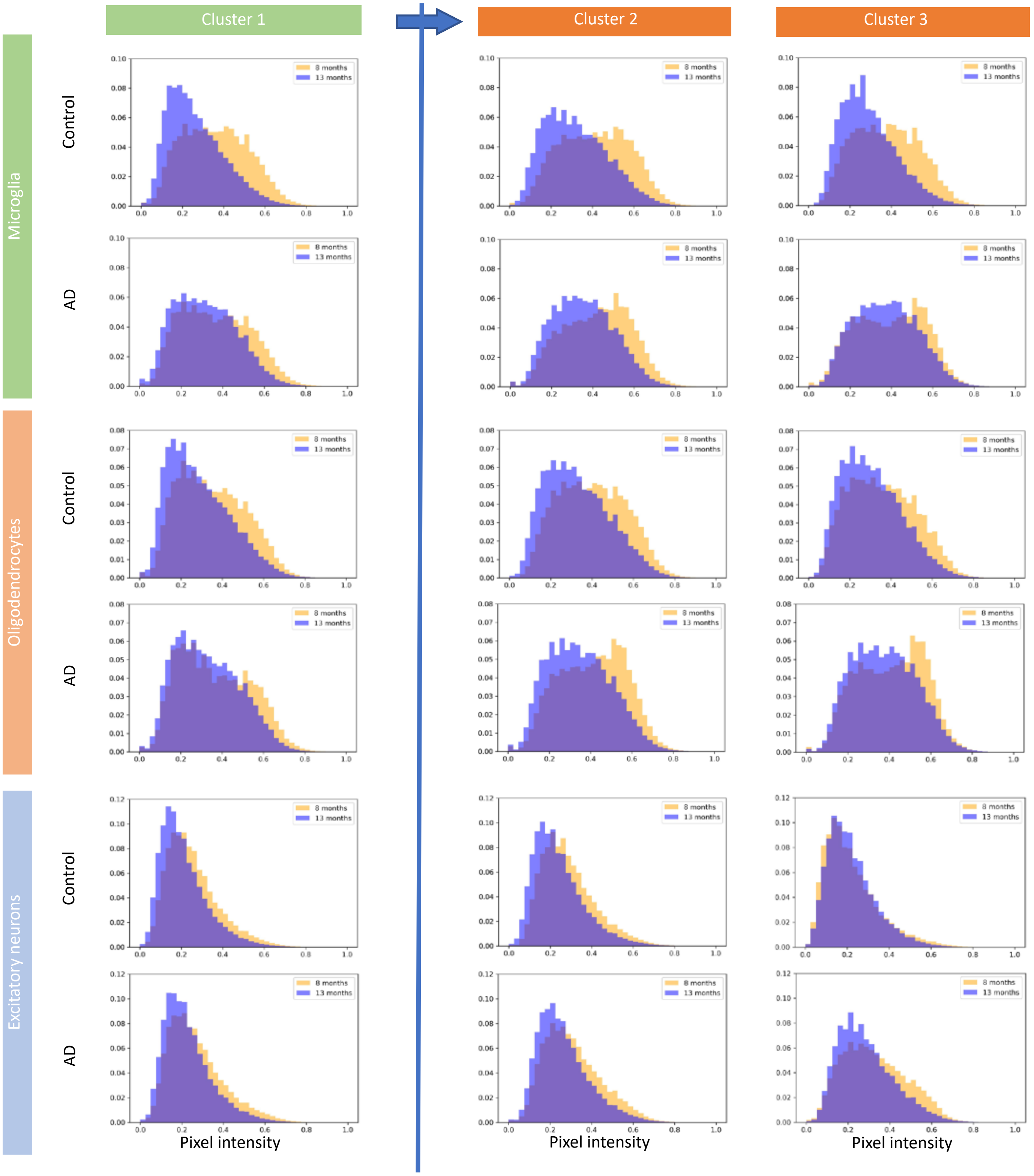

Supplementary Figure 26

Supplementary Figure 26. Histograms of chromatin pixel intensities in all cells of each cell type in the specific cortex cluster at each time point, normalized to sum to 1.

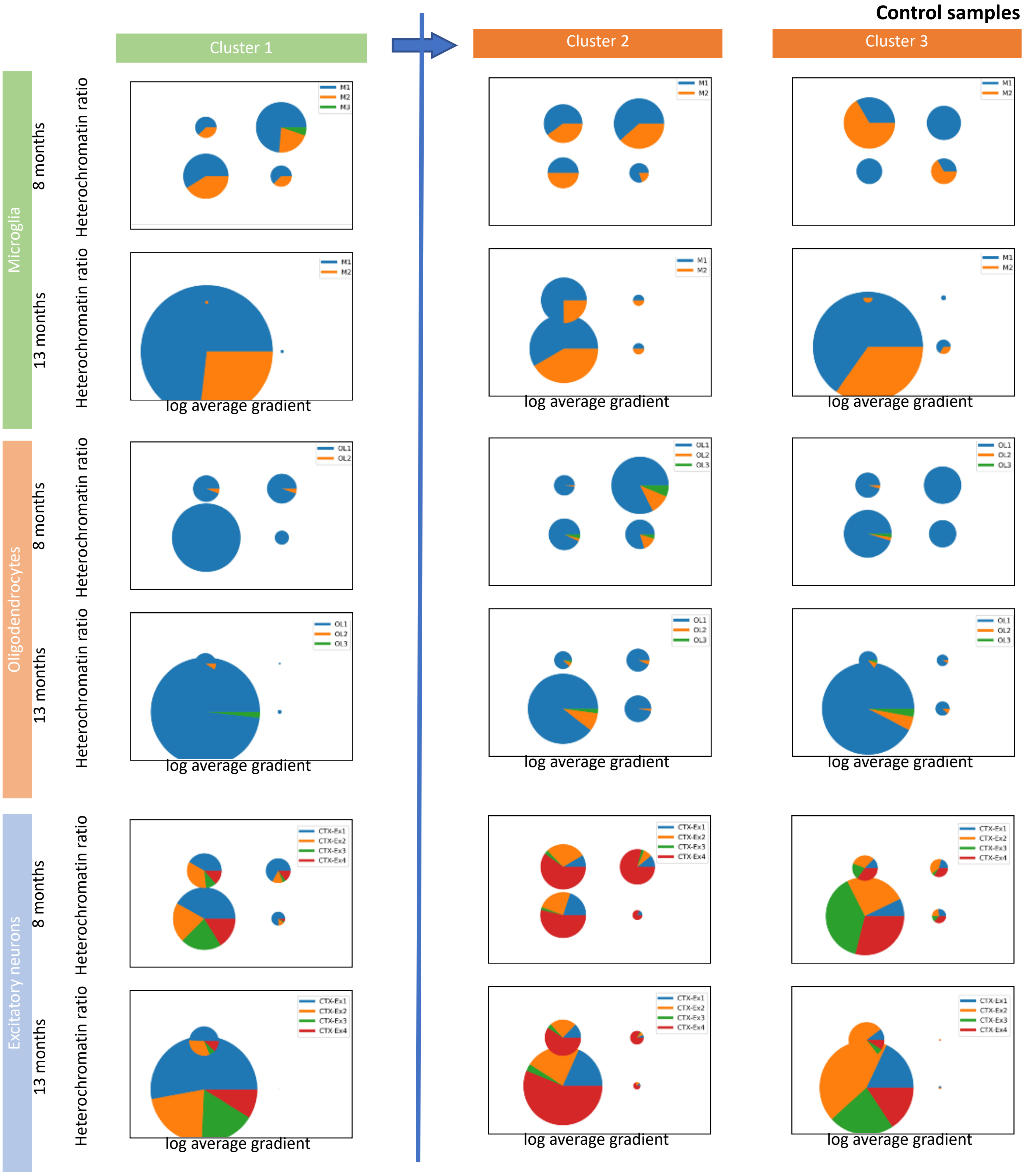

Supplementary Figure 27

Supplementary Figure 27. Cells are grouped into four quadrants by heterochromatin ratio (threshold at 0.5 for excitatory neurons and 0.8 for others) and log average gradient (threshold at 6.5 for excitatory neurons and 6.7 for others). The sizes of the pie charts are proportional to the fraction of cells in each quadrant with respect to all four quadrants in the same clusters. The angles in the pie charts are proportional to the fraction of each subtype in each quadrant.

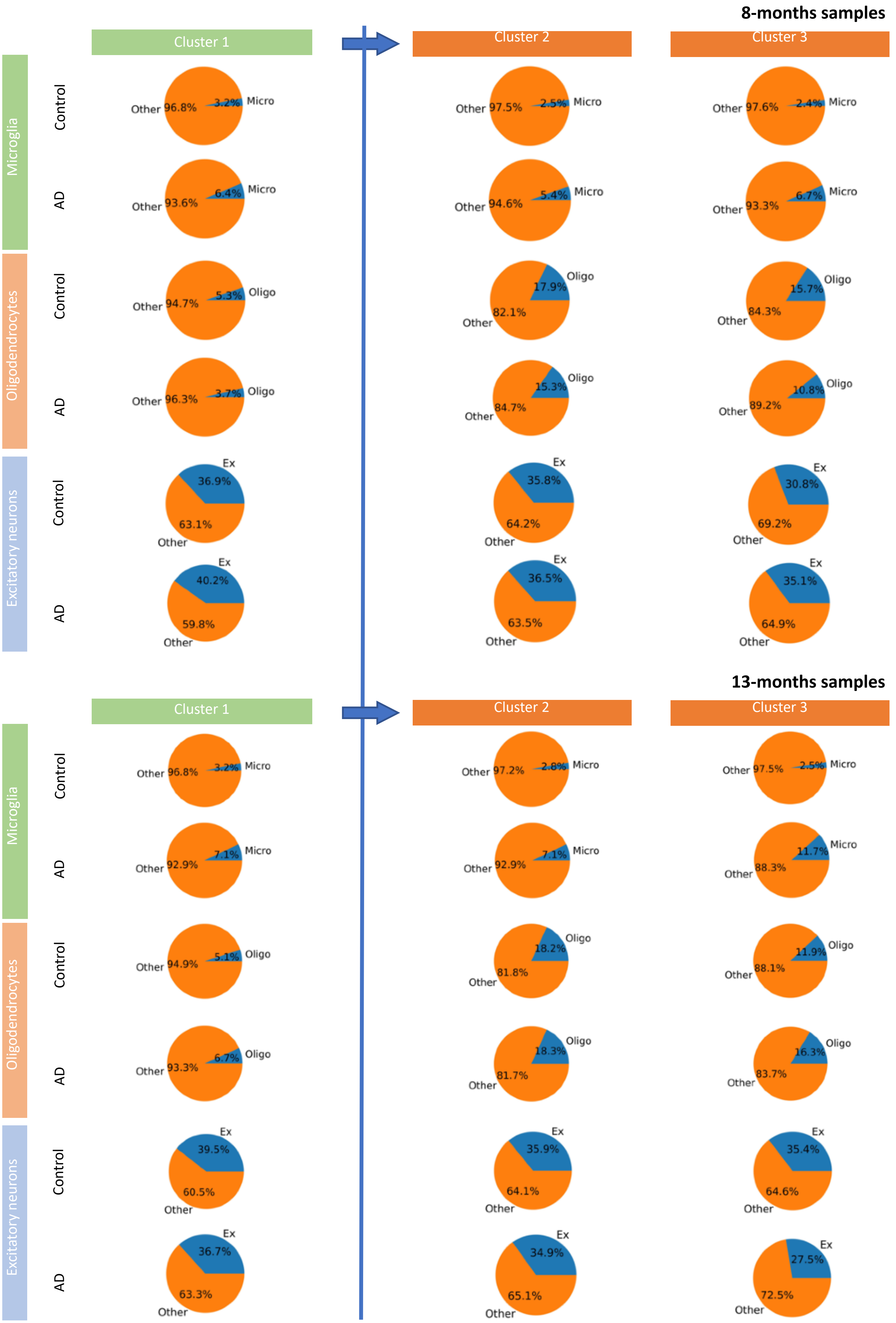

Supplementary Figure 28

Supplementary Figure 28. Fractions of microglia (Micro), oligodendrocytes (Oligo), and excitatory neurons (Ex) among all cell types in each cluster.

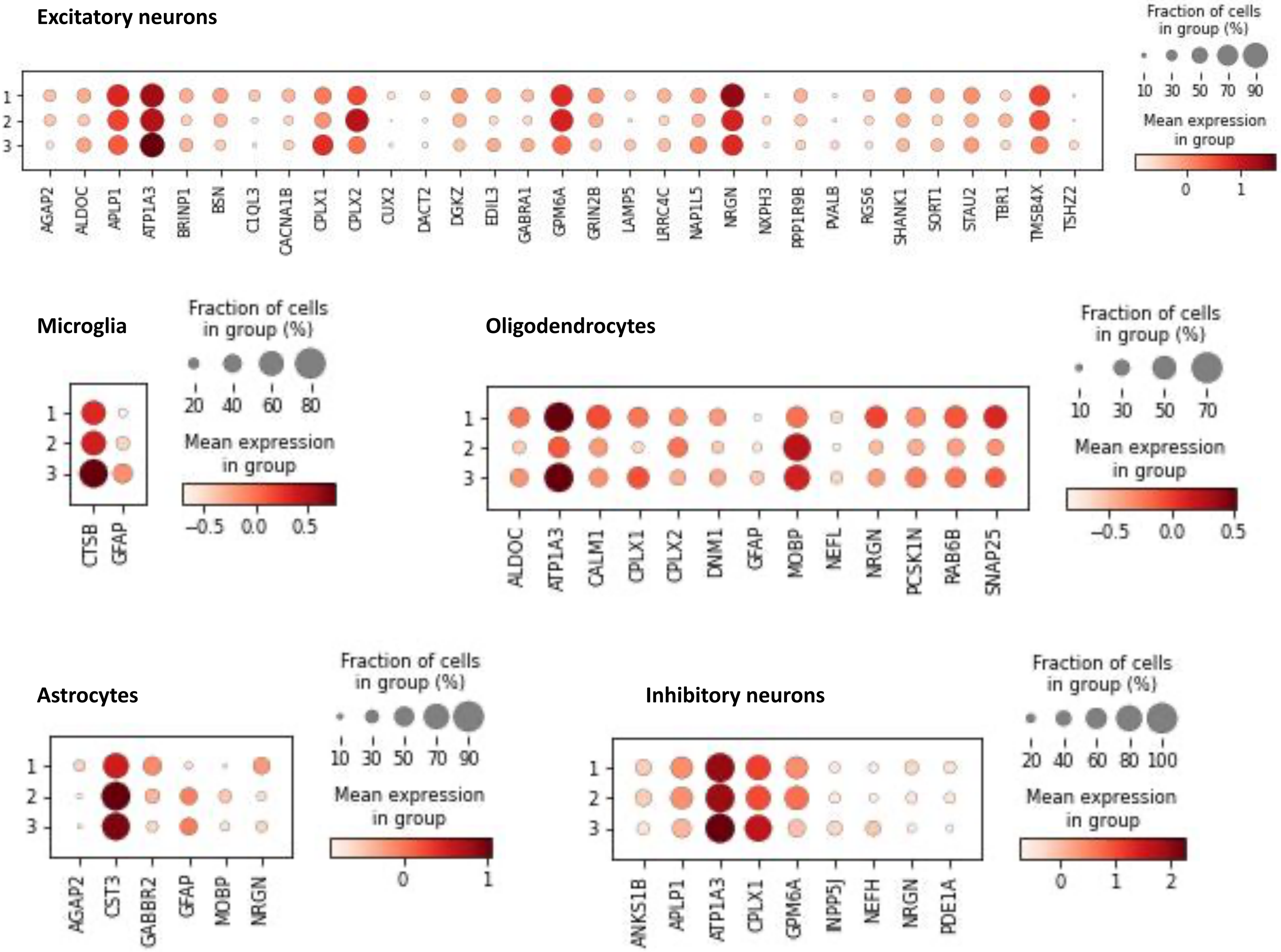

Supplementary Figure 29

Supplementary Figure 29. Differential expression analysis of the three cortex regions identified by STACI.

Statistical significance was defined as p-value < 0.05 after correction by Benjamini-Hochberg procedure[8] and fold change of at least 10% in either direction. Raw p-values were calculated using the Wilcoxon rank-sum test. At most 15 differentially expressed genes with smallest p-values are shown. The exact p-values of all differentially expressed genes are provided in Supplementary Dataset 2.

References

1. Zeng, H. *et al.* Integrative in situ mapping of single-cell transcriptional states and tissue histopathology in an Alzheimer's disease model. *bioRxiv* (2022).
2. Multiomic Integration Neuroscience Application Note: Visium for FFPE Plus Immunofluorescence Alzheimer's Disease Mouse Model Brain Coronal Sections from One Hemisphere Over a Time Course. *10x Genomics* https://www.10xgenomics.com/resources/datasets/multiomic-integration-neuroscience-application-note-visium-for-ffpe-plus-immunofluorescence-alzheimers-disease-mouse-model-brain-coronal-sections-from-one-hemisphere-over-a-time-course-1-standard.
3. Pham, D. *et al.* stLearn: integrating spatial location, tissue morphology and gene expression to find cell types, cell-cell interactions and spatial trajectories within undissociated tissues. 2020.05.31.125658 Preprint at https://doi.org/10.1101/2020.05.31.125658 (2020).
4. Zhu, Q., Shah, S., Dries, R., Cai, L. & Yuan, G.-C. Identification of spatially associated subpopulations by combining scRNAseq and sequential fluorescence in situ hybridization data. *Nat Biotechnol* **36**, 1183–1190 (2018).
5. Bao, F. *et al.* Integrative spatial analysis of cell morphologies and transcriptional states with MUSE. *Nat Biotechnol* 1–10 (2022) doi:10.1038/s41587-022-01251-z.
6. Haghverdi, L., Lun, A. T. L., Morgan, M. D. & Marioni, J. C. Batch effects in single-cell RNA sequencing data are corrected by matching mutual nearest neighbours. *Nat Biotechnol* **36**, 421–427 (2018).
7. Lein, E. S. *et al.* Genome-wide atlas of gene expression in the adult mouse brain. *Nature* **445**, 168–176 (2007).
8. Benjamini, Y. & Hochberg, Y. Controlling the False Discovery Rate: A Practical and Powerful Approach to Multiple Testing. *Journal of the Royal Statistical Society. Series B (Methodological)* **57**, 289–300 (1995).
