# Supplementary material for: Graph-based autoencoder integrates spatial transcriptomics with chromatin images and identifies joint biomarkers for Alzheimer’s disease
Source: Nat Commun. 2022 Dec 3;13:7480. doi: 10.1038/s41467-022-35233-1 (PMC9719477; doi:10.1038/s41467-022-35233-1)
Supplement: Supplementary file 4 — Description of Additional Supplementary Files [file 41467_2022_35233_MOESM4_ESM.pdf]

Title: Supplementary Data 1.
Description: Classification loss of plaque size regression as fraction of misclassified cells. The classification error for each of the regression models was calculated as described in Methods.
